# Rigorous nested cross-validation and external generalization-gap analysis of a machine-learning classifier for U-251 glioblastoma cytotoxicity

**DOI:** 10.1371/journal.pone.0361912

**Published:** 2026-11-03

**Authors:** Yuki Uemichi, Shuhei Nakao, Shuji Nagano, Miyuki Mabuchi, Yuya Kawabata, Yoshihiro Uesawa, Tadashi Shimizu

**Affiliations:** 1 School of Pharmacy, Hyogo Medical University, Kobe, Hyogo, Japan; 2 Department of Medical Molecular Informatics, Meiji Pharmaceutical University, Kiyose, Tokyo, Japan; Universidad El Bosque, COLOMBIA

## Abstract

Glioblastoma remains difficult to treat, and drug-repositioning strategies require efficient methods to evaluate chemical libraries. Here, we developed a machine-learning binary classifier for compound activity against U-251 glioblastoma cells using a corrected single-center dataset of 236 compounds evaluated under a standardized cell-viability assay. Compounds were labeled as active or inactive using a predefined viability criterion (<50% vs. ≥ 50%) within a single-point screening framework using 10 µM as the upper screening concentration. To reduce optimistic bias and information leakage, we used repeated nested cross-validation in which preprocessing, feature selection, and hyperparameter optimization were performed within the training folds. ExtraTrees was selected based on Matthews correlation coefficient, showing moderate internal performance (ROC-AUC, 0.76; balanced accuracy, 0.66; MCC, 0.36). Structural analyses using nearest-neighbor Tanimoto similarity and Bemis–Murcko scaffolds indicated that the training set was scaffold-diverse with limited series bias. However, when the locked model was applied to an independent external set of 53 compounds, all compounds were classified as inactive, resulting in zero sensitivity, balanced accuracy of 0.5, and MCC of 0. The external ROC-AUC based on continuous prediction scores was 0.37, indicating poor threshold-independent ranking performance. Nearest-neighbor Tanimoto similarity did not explain prediction correctness or rescue active-compound recovery. Y-randomization supported the presence of a non-random internal signal, whereas scaffold-grouped cross-validation suggested that internal ROC-AUC was not solely driven by scaffold leakage. Overall, this study provides a reusable dataset and reproducible workflow, and highlights a substantial generalization gap between internal validation and external prediction in small chemical machine-learning datasets.

## 1. Introduction

Glioblastoma is the most aggressive and lethal type of brain tumor, with a five-year survival rate of approximately 7% [1]. Because glioblastoma is highly infiltrative, complete surgical resection is rarely achievable, and recurrence is common. Chemotherapy also remains challenging: delivery of many anticancer agents to the brain is limited by the blood–brain barrier (BBB), and treatment resistance further restricts clinical benefit [2]. Temozolomide, a key chemotherapeutic agent used in current clinical practice, can reach the brain; however, its efficacy is often reduced by intrinsic or acquired resistance mechanisms, including the expression of DNA repair enzymes such as *O*^6^-methylguanine-DNA methyltransferase (MGMT) [3,4]. Given the limited number of effective therapeutic options, there is an urgent need to discover new candidates for glioblastoma treatment.

Drug repositioning refers to the identification of new therapeutic applications for existing approved drugs or previously discontinued compounds, repurposing them for diseases beyond their original indications [5,6]. This strategy can shorten development timelines by leveraging prior knowledge on pharmacology and safety. In our previous study, we demonstrated that blonanserin and lomerizine, both capable of crossing the BBB, effectively inhibited the growth of glioblastoma cells [7,8]. However, a comprehensive evaluation of the therapeutic potential of tens of thousands of approved drugs, withdrawn products, and their metabolites remains a major challenge, requiring extensive time, financial investment, and human resources. Therefore, practical repositioning efforts benefit from methods that can prioritize compounds for follow-up testing.

Machine learning (ML), a subset of artificial intelligence, has been increasingly applied to construct quantitative structure–activity relationship (QSAR) models that predict biological activity from chemical features [9–13]. Public databases such as ChEMBL [14] and PubChem [15] provide large-scale assay data with broad chemical diversity and are valuable resources for benchmarking and model development. Several predictive models of compound activity against glioblastoma cells have also been developed using public resources such as ChEMBL [16], which provide substantial scale and chemical diversity and greatly simplify dataset assembly. Nevertheless, heterogeneity in assay protocols, including exposure time, concentration range, readout method, and endpoint definition, can introduce label noise and complicate model interpretation when data from multiple sources are aggregated. In contrast, in-house datasets generated under harmonized experimental conditions can reduce inter-assay variability, although they are often smaller, more imbalanced, and less chemically comprehensive.

These characteristics create an important methodological challenge. Models trained on small, standardized in-house datasets may show apparently favorable internal cross-validation performance, but it remains unclear whether such performance reflects a transferable structure–activity relationship or a signal that is largely confined to the local chemical space represented in the training set. This issue is particularly relevant in drug-repositioning studies, where a model may be expected to support the evaluation of structurally diverse external compounds. Therefore, rigorous internal validation alone is insufficient; external validation, applicability-domain assessment, calibration analysis, and tests for potential overfitting are needed to clarify the extent and limits of model generalizability.

In this study, we developed an ML-based binary classification model to distinguish compounds as “active” or “inactive” against U-251 glioblastoma cells under a predefined, standardized cell-viability assay condition, using a corrected single-center dataset. Rather than presenting the model as a ready-to-use screening or compound-prioritization tool, we aimed to examine whether an internally validated classifier trained on a small and imbalanced chemical dataset could retain predictive performance on independent external compounds. Specifically, we used leakage-controlled repeated nested cross-validation to estimate internal performance and complemented this analysis with external validation, Y-randomization, scaffold-aware validation, calibration analysis, and applicability-domain analyses. Through these analyses, this study provides a reproducible dataset and modeling workflow, and serves as a cautionary generalization-gap benchmark for evaluating the discrepancy between internal cross-validation performance and external generalizability in small chemical ML studies.

## 2. Methods

### 2.1. Chemistry

Reagents and solvents used for compound synthesis were purchased from commercial sources and used without further purification. Thin-layer chromatography (Silica gel 60 F254, Merck, 1.05715, Darmstadt, Germany) was employed to monitor the reaction progress with UV detection at 254 nm. Silica gel 60N (Kanto Chemical, 37561−85, Chuo-ku, Tokyo, Japan) was used for purification by column chromatography. ^1^H-NMR and ^13^C-NMR spectra were recorded on a JEOL JNM-ECZL400S. ESI-MS was taken on a SHIMADZU LCMS-9030. Detailed information on each compound, including its source (commercial supplier or in-house synthesis) and, where applicable, the corresponding synthesis method, is provided in the Supporting Information (S1 File, S2 File).

### 2.2. Cell culture

Human glioblastoma cell line (U-251) was obtained from Dr. T. Sasayama (Kobe University, Japan). The cells were cultured in Dulbecco’s modified Eagle’s medium (Fujifilm Wako, 043−30085, Osaka-shi, Osaka, Japan) supplemented with 10% fetal bovine serum (FBS; Biosera, FB-1360/500, Cholet, France). The cells were grown in an incubator at 37°C under 5% CO_2_ and subcultured at 80% confluence using trypsin-ethylenediaminetetraacetic acid (Nacalai tesque, 35553−74, Kyoto-shi, Kyoto, Japan).

### 2.3. Cell assay

U-251 cells were seeded at a density of 3,000 cells/well in a 96-well plate (100 μL) and incubated at 37°C for 24 h. Subsequently, the cells were treated with the test compounds and incubated at 37°C for 48 h. The standard screening concentration was 10 µM; however, a small subset of compounds was evaluated at 5 µM or 0.5 µM, as detailed in S1 Table. The final DMSO concentration was 0.1%. Cell viability was determined using Cell Counting Kit-8 (Dojindo, CK04, Kamimashiki-gun, Kumamoto, Japan), according to the instructions provided by the manufacturer (n = 3 or 4). After 2 h, absorbance was measured at 450 nm using VersaMax (Molecular Devices, San Jose, California, USA). Cell viability was calculated by normalizing the absorbance of each treated group to that of the vehicle control containing 0.1% DMSO, which was set at 100%.

### 2.4. Calculation of descriptors for assay compounds

The simplified molecular input line entry system (SMILES) strings of the compounds were obtained from PubChem. For compounds that were synthesized in the laboratory and not registered in PubChem, the SMILES were obtained from ChemDraw Prime 19.1 (PerkinElmer, Bridgeport Avenue, Shelton, USA). The SMILES strings of the compounds were converted to canonical SMILES, and 4179 descriptors derived from the 2D structure were calculated using alvaDesc (alvascience, Via Giuseppe Parini, Lecco, Italy).

### 2.5. Machine-learning workflow and validation scheme

We built binary classification models to predict compound activity against human glioblastoma U-251 cells using an in-house dataset (n = 236). The response variable was defined as a binary operational screening label using 10 µM as the upper screening concentration. Compounds showing U-251 cell viability <50% after 48 h exposure at 10 µM (i.e., > 50% inhibition relative to the vehicle control) were labeled active (1), whereas those showing viability ≥50% (i.e., ≤ 50% inhibition) were labeled inactive (0). For a small subset of compounds that had been evaluated at lower concentrations (5 µM or 0.5 µM), compounds showing cell viability <50% at these concentrations were also classified as active because they met the predefined activity criterion at a concentration below the 10 µM screening cutoff. The specific assay concentration used for each compound is provided in S1 Table. The terms “active” and “inactive” are operational labels defined solely by U-251 cell viability under the specified screening condition and do not indicate therapeutic efficacy, potency, or tumor selectivity.

All analyses were implemented in Python (Jupyter Notebook) using scikit-learn and imbalanced-learn. To obtain an unbiased estimate of generalization performance and avoid optimistic “lucky split” artifacts, we employed repeated nested cross-validation. The outer loop used repeated stratified K-fold splits (5-folds × 10 repeats; 50 outer test splits) for performance estimation. For each outer training split, hyperparameters were optimized in the inner loop using stratified K-fold cross-validation (4 folds) with RandomizedSearchCV (30 iterations per model). The primary optimization metric in the inner loop was average precision (PR-AUC).

All preprocessing steps were performed within the pipeline and were fitted using training folds only to prevent information leakage. Briefly, non-feature columns (e.g., ID/SMILES/label columns) were removed, remaining descriptor columns were coerced to numeric values, and features were then processed as follows: (i) removal of descriptors with high missingness, (ii) removal of descriptor columns exhibiting frequent extreme values using a robust z-score based on the median absolute deviation (MAD), (iii) median imputation for missing values, (iv) removal of near-constant features using a variance threshold, (v) filtering of highly correlated features (absolute correlation threshold), and (vi) univariate feature selection using SelectKBest with mutual information, where the number of selected features (k) was treated as a tunable hyperparameter. Several preprocessing hyperparameters (e.g., missingness threshold, outlier filter parameters, variance threshold, correlation threshold, and k) were included in the inner-loop hyperparameter search.

We evaluated multiple algorithms including logistic regression (LogReg), RBF-kernel support vector machine (SVM_RBF), random forest (RF), extremely randomized trees (ExtraTrees), gradient boosting decision trees (GBDT), and multilayer perceptron (MLP). Class-weighting options were also considered as part of hyperparameter search for applicable models. For each algorithm, outer-loop predictions were stored and performance metrics were computed on each outer test split, and summary results were reported as the mean ± 95% confidence interval across the 50 outer splits. The best-performing algorithm was selected based on the mean Matthews correlation coefficient (MCC) across the outer cross-validation splits, and a final model was refitted on the full dataset using the same inner-loop search procedure. For the final model, the classification threshold was determined by maximizing Youden’s J statistic using prediction scores obtained from the full training dataset (0.707815). This threshold was then fixed and applied to the independent external validation set. The Python code described above has been published on Zenodo (https://doi.org/10.5281/zenodo.20781177).

### 2.6. Performance evaluation on the outer cross-validation splits

Model performance was evaluated on the outer test folds of the repeated nested cross-validation (5-folds × 10 repeats; 50 outer splits). For each outer split, predicted scores were obtained for the held-out test fold, and the following performance metrics were calculated: the area under the ROC curve (ROC-AUC) and average precision (AP), accuracy (ACC), F1-score (F1), balanced accuracy (BA), and Matthews correlation coefficient (MCC).

The best model was selected based on MCC. To summarize performance across repeated outer splits, each metric was reported as the mean and 95% confidence interval (CI) across the 50 outer splits. The 95% CI was computed from the distribution of split-wise metric values, thereby reflecting variability due to data partitioning. For transparency, aggregated confusion-matrix counts across outer splits can be provided as descriptive summaries; however, inferential statements are based on the split-wise mean ± 95% CI. ROC-AUC and AP were calculated using continuous predicted scores and were therefore treated as threshold-independent measures of ranking performance. In contrast, accuracy, F1-score, balanced accuracy, and MCC were calculated from thresholded predicted class labels.

### 2.7. Structural similarity analysis and applicability domain based on ECFP and Tanimoto similarity

Structural similarity between compounds in the training set and an external compound set was quantified using Morgan fingerprints (ECFP) and Tanimoto similarity. Compound structures were provided as SMILES strings in Excel files and converted to RDKit molecule objects; invalid or empty SMILES entries were excluded from subsequent analyses. For each valid molecule, Morgan fingerprints were generated as binary bit vectors using RDKit (radius = 2; 1024 bits).

Tanimoto similarities were computed using RDKit’s bulk similarity routine. To summarize similarity relationships, we calculated nearest-neighbor (NN) similarity in two ways. First, for training set, the within-set NN similarity for each compound was defined as the highest Tanimoto similarity to any other compound in the same dataset (self-comparisons excluded). Second, for each external compound, the external-to-training NN similarity was defined as the highest Tanimoto similarity to any compound in the training set, thereby quantifying proximity of each external compound to the training chemical space.

### 2.8. Bemis–Murcko scaffolds–based structural diversity analysis

Scaffold-level structural diversity was assessed using Bemis–Murcko scaffolds. Compound structures were provided as SMILES strings in an Excel file and converted to RDKit molecule objects; invalid or empty SMILES entries were excluded from the analysis. For each valid molecule, the Bemis–Murcko scaffold was extracted using the RDKit scaffold utility and represented as a scaffold SMILES string. Compounds for which the scaffold could not be generated were also excluded.

Scaffold frequencies were calculated by counting the occurrence of each unique scaffold. Structural diversity and series bias were summarized using the following scaffold-level statistics: the number of compounds used for the analysis, the number of unique scaffolds, the scaffold ratio (unique scaffolds divided by the number of compounds), the Top-1 and Top-5 scaffold shares (fractions of compounds belonging to the most frequent scaffold and to the five most frequent scaffolds), and the normalized Shannon entropy of the scaffold frequency distribution (0–1 scale, where values closer to 1 indicate a more even distribution of scaffolds).

### 2.9. Supplementary validation and statistical analysis of model behavior

To further assess the robustness, transferability, and applicability-domain characteristics of the final model, we performed a series of supplementary statistical analyses using Python, scikit-learn, RDKit, NumPy, pandas, SciPy, and statsmodels. These analyses were conducted as post hoc evaluations of model behavior and were not used for model selection or hyperparameter optimization.

Prediction correctness was analyzed as a function of nearest-neighbor Tanimoto similarity using logistic regression. Among experimentally active external compounds, active recovery was also examined in relation to nearest-neighbor similarity when variation in recovery status was present. Spearman rank correlations were used to assess associations between model prediction scores and measured cell viability, as well as between prediction scores and experimental activity labels.

To test whether the internal classification signal could be explained by random label assignment, Y-randomization was performed using label permutation analysis. A fixed ExtraTrees-based validation workflow was used for this supplementary analysis. Before modeling, molecular descriptors were subjected to label-independent preprocessing, including removal of descriptors with more than 20% missing values, median imputation, removal of near-constant descriptors, and greedy removal of highly correlated descriptors with an absolute correlation coefficient greater than 0.95. The classifier pipeline consisted of SelectKBest feature selection based on mutual information followed by an ExtraTrees classifier with balanced class weights. Five-fold stratified cross-validation was used to calculate ROC-AUC for the original labels and for randomly permuted labels. The empirical p-value was calculated from 1000 label permutations.

To examine whether internal model performance was driven by scaffold-level leakage, random 5-fold cross-validation was compared with leave-scaffold-out grouped cross-validation. Bemis–Murcko scaffolds were generated from SMILES strings using RDKit and used as grouping labels. In the leave-scaffold-out analysis, compounds sharing the same scaffold were not allowed to appear in both training and test folds. The absence of scaffold leakage across folds was confirmed computationally. Out-of-fold prediction scores were used to calculate ROC-AUC, AP, MCC, balanced accuracy, sensitivity, and specificity for both random and scaffold-grouped cross-validation.

To summarize the internal-to-external generalization gap, the performance metrics from random cross-validation, leave-scaffold-out cross-validation, and the locked external validation set were compiled into a single comparison table. The overall probabilistic predictive performance of the locked model on the external set was evaluated using the Brier score. The distribution of external scores was also compared with the predefined final decision threshold.

Finally, covariate shift and applicability-domain coverage were evaluated using nearest-neighbor Tanimoto similarity. The within-training nearest-neighbor similarity distribution was calculated by comparing each training compound with all other training compounds. The 5th percentile of this within-training distribution was used as an empirical low-similarity applicability-domain threshold. External compounds were then characterized by their nearest-neighbor similarity to the training set, the fraction falling below this empirical threshold, and the fractions with nearest-neighbor similarities below 0.3 or above 0.5 and 0.7.

### 2.10. AI-Assisted Python Code Generation

ChatGPT (GPT-5.5, OpenAI) was used to assist in generating Python code for the analyses performed in this study. The authors reviewed and executed the AI-assisted code and verified that the resulting outputs were consistent with the results reported in this article.

## 3. Results

The U-251 cell viability results for the 236 compounds are shown in Fig 1. Most compounds were evaluated at 10 µM, whereas a small subset was evaluated at 5 µM or 0.5 µM, as detailed in S1 Table. Of the 236 compounds evaluated, 45 met the predefined activity criterion of cell viability <50%, whereas 191 showed cell viability ≥50%.

**Fig 1:**
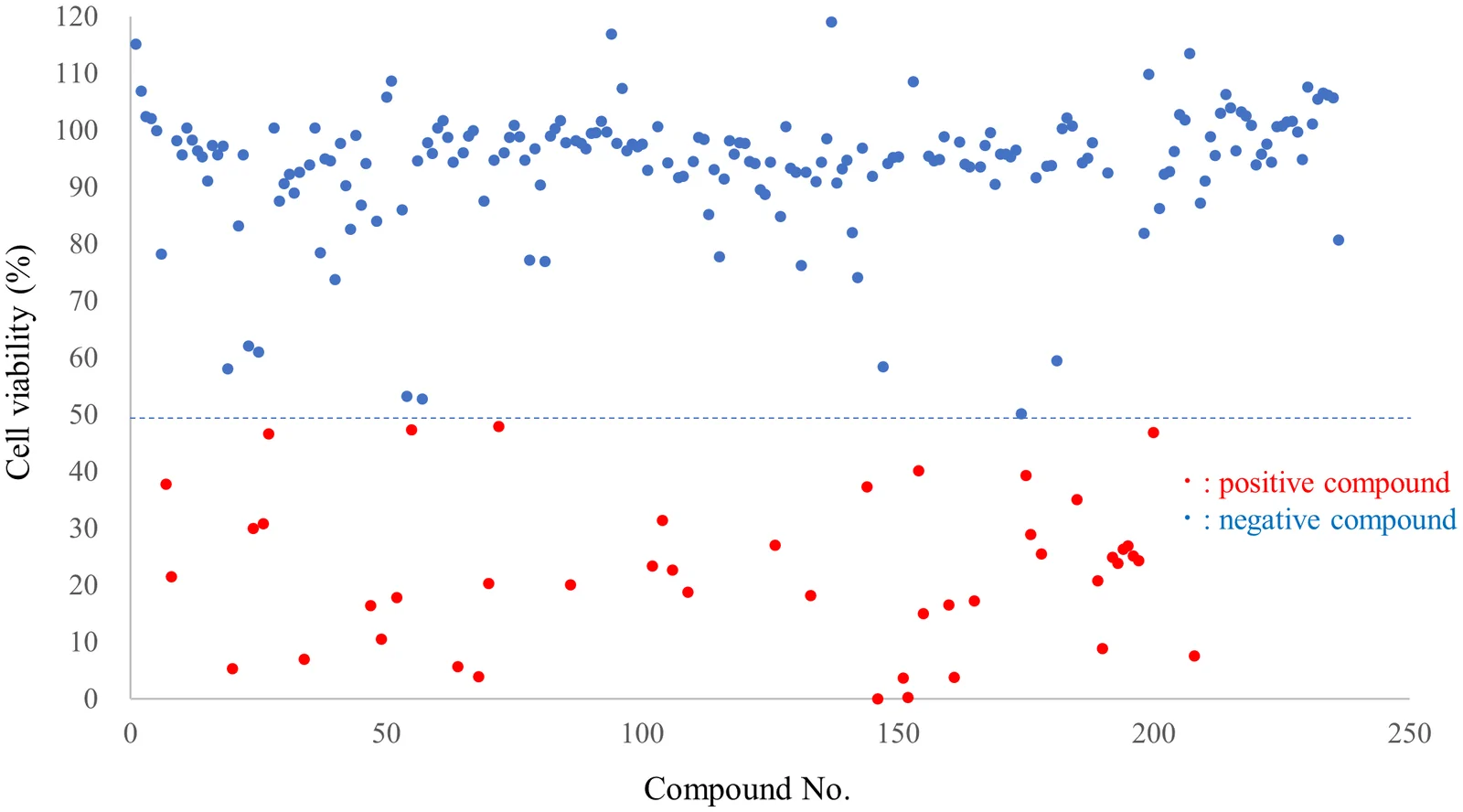
Cell viability after treatment with each compound. The vertical axis represents cell viability and the horizontal axis represents the compound number. The compound numbers are listed in S1 Table. Blue dots indicate compounds with cell viability of 50% or higher, and red dots indicate compounds with cell viability lower than 50%. Most compounds were evaluated at 10 µM; a small subset was evaluated at 5 µM or 0.5 µM, as specified in S1 Table (48 h, WST-8, n = 3-4).

Using the nested cross-validation framework, hyperparameters were optimized within the inner cross-validation loop for each outer training fold, and each optimized model was evaluated on the corresponding held-out outer test fold. Performance metrics derived from the confusion matrices were calculated for each algorithm, as summarized in Table 1. The aggregated confusion matrices across all outer test folds are shown in S2 Table. Because the dataset used for model training was class-imbalanced, we placed particular emphasis on the Matthews correlation coefficient (MCC), which is suitable for evaluating binary classification performance under imbalanced class distributions. Based on the highest MCC in the outer cross-validation evaluation, ExtraTrees was selected as the best-performing model. After model-family selection, the final ExtraTrees model was refitted using the full training dataset, and the selected hyperparameters of this final model are summarized in Table 2.

**Table 1:** Performance metrics (mean and 95%CI) of each algorithm.

Algorithm	ROC-AUC (95%CI)	AP (95%CI)	BA (95%CI)	MCC (95%CI)	F1 (95%CI)	ACC (95%CI)
ExtraTrees	0.76 (0.73-0.79)	0.57 (0.54-0.61)	0.66 (0.63-0.68)	0.36 (0.31-0.40)	0.43 (0.38-0.47)	0.81 (0.80-0.82)
RF	0.76 (0.74-0.78)	0.56 (0.52-0.59)	0.65 (0.62-0.67)	0.34 (0.30-0.39)	0.42 (0.38-0.46)	0.81 (0.79-0.82)
GBDT	0.77 (0.74-0.79)	0.57 (0.53-0.60)	0.59 (0.57-0.60)	0.31 (0.27-0.35)	0.28 (0.24-0.33)	0.83 (0.82-0.84)
SVM_RBF	0.74 (0.72-0.77)	0.54 (0.50-0.57)	0.63 (0.61-0.66)	0.25 (0.20-0.30)	0.37 (0.31-0.42)	0.73 (0.71-0.76)
LogReg	0.68 (0.66-0.70)	0.43 (0.39-0.46)	0.63 (0.61-0.65)	0.24 (0.20-0.28)	0.40 (0.38-0.43)	0.69 (0.66-0.72)
MLP	0.66 (0.63-0.69)	0.42 (0.39-0.46)	0.63 (0.61-0.66)	0.23 (0.19-0.28)	0.39 (0.35-0.42)	0.68 (0.65-0.72)

**Table 2:** The selected hyperparameters of the final model (ExtraTrees).

Parameter	Selected value
Maximum allowed missing-value fraction	0.30
Robust z-score threshold for outlier detection	6.0
Maximum allowed outlier fraction	0.002
Variance threshold	1 x 10^−6^
Correlation threshold	0.98
Number of selected features	25
Number of trees	800
Maximum tree depth	10
Number of features considered at each split	sqrt
Minimum samples required to split an internal node	2
Minimum samples required at a leaf node	1
Class weighting	balanced
Final classification threshold	0.707815

To evaluate the structural diversity of the compounds used for model training, NN Tanimoto similarity and Bemis–Murcko scaffold analyses were performed, and the results are shown in Fig 2 and Table 3, respectively. An NN Tanimoto similarity value close to 1 indicates that a structurally similar compound was present in the dataset, whereas a value close to 0 indicates low structural similarity to all other compounds in the dataset. In the distribution of within-training NN Tanimoto similarities, the most frequent range was 0.2–0.3, which included 89 compounds. Overall, the within-training NN Tanimoto similarities were relatively low, with a median of 0.33, 25th percentile of 0.26, 75th percentile of 0.54, and interquartile range (IQR) of 0.28. For each compound, the NN Tanimoto similarity was defined as the maximum Tanimoto similarity to any other compound in the training dataset, excluding self-comparisons.

**Table 3:** Summary of Bemis–Murcko scaffold analysis of the training compound set.

Parameter	Value
Number of compounds analyzed	234 ^*)^
Number of unique scaffolds	189
Scaffold ratio	0.81
Top1 scaffold share	0.06
Top5 scaffold share	0.15
Normalized Shannon entropy	0.96

*) Two compounds were excluded from the Bemis–Murcko scaffold analysis because their SMILES strings could not be parsed into valid RDKit molecule objects or yielded empty Bemis–Murcko scaffold representations.

**Fig 2:**
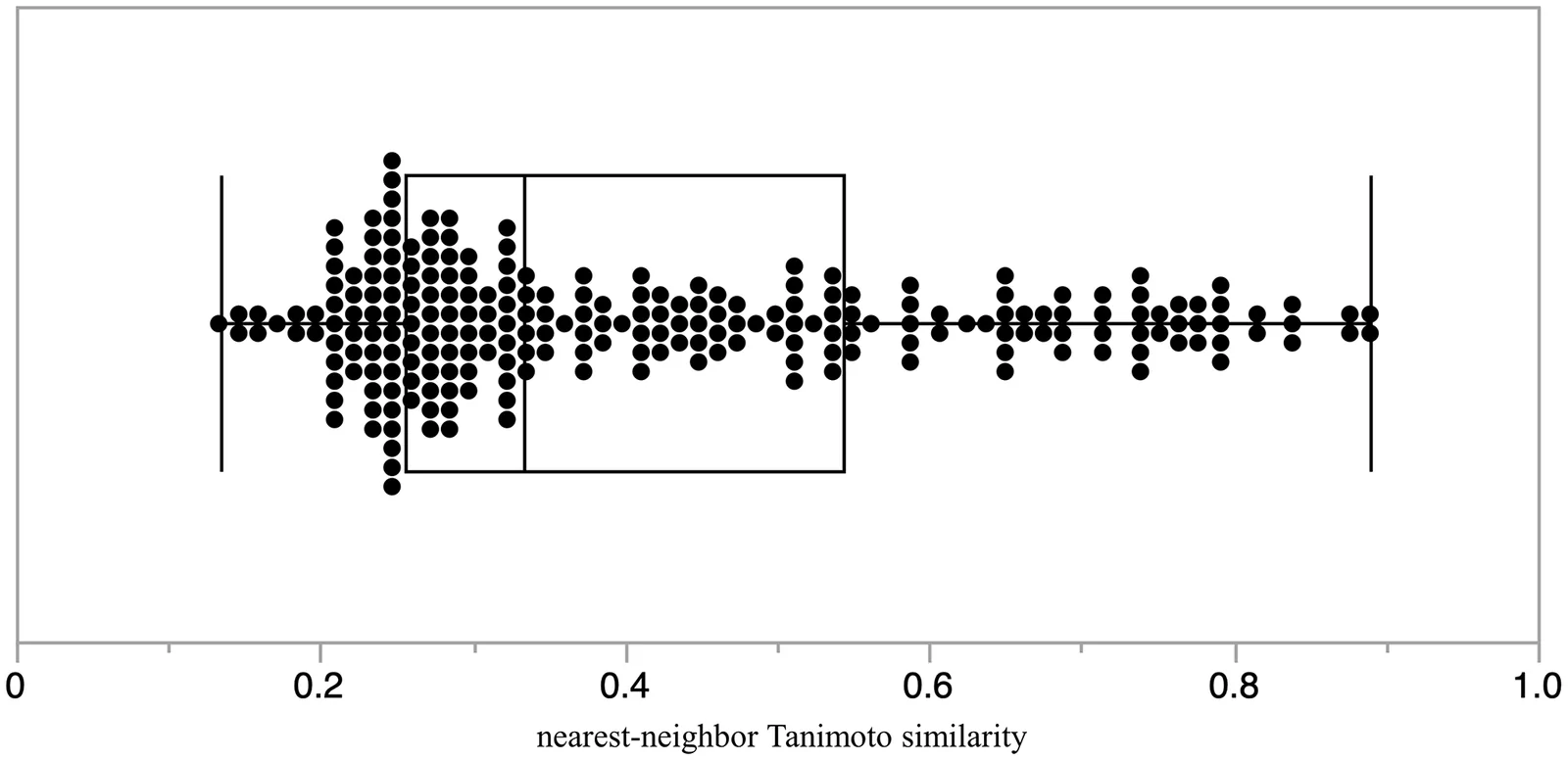
Distribution of nearest-neighbor (NN) Tanimoto similarity in the training compound set. NN Tanimoto similarity was calculated for each compound in the training set using Morgan fingerprints (radius = 2, 1024 bits). For each compound, the NN Tanimoto similarity was defined as the maximum Tanimoto similarity to any other compound in the training set, excluding self-comparisons. The box plot summarizes the distribution of NN Tanimoto similarity values across compounds. Lower values indicate lower structural similarity to all other compounds in the training set, whereas higher values indicate the presence of structurally similar compounds. (median of 0.33, 25th percentile of 0.26, 75th percentile of 0.54, and interquartile range (IQR) of 0.28).

Bemis–Murcko scaffold analysis further supported the scaffold-level structural diversity of the training compound set (Table 3). Among the 234 compounds included in this analysis, 189 unique Bemis–Murcko scaffolds were identified, resulting in a scaffold ratio (number of unique scaffolds/number of compounds) of 0.81. The most frequent scaffold accounted for only 6% of the compounds (Top1 share = 0.06), and the cumulative share of the top five scaffolds was 15% (Top5 share = 0.15). In addition, the normalized Shannon entropy of the scaffold frequency distribution was 0.96 on a 0–1 scale.

The final model was applied to an independent small-scale compound library consisting of 53 compounds that had not been used for model training, and the predicted labels were compared with the experimental results. The external validation performance was as follows: sensitivity = 0, specificity = 1.00, positive predictive value (PPV) = NA (no compound predicted active), negative predictive value (NPV) = 0.62, balanced accuracy (BA) = 0.5, Matthews correlation coefficient (MCC) = 0, accuracy (ACC) = 0.62, ROC-AUC = 0.37, AP = 0.32 (Table 4). The experimentally determined cell viability values for these compounds are shown in S3 Table. We next examined the structural proximity of the 53 external compounds to the training set using NN Tanimoto similarity (Fig 3). The NN Tanimoto similarity values were generally low, with a median of 0.23, 25th percentile of 0.21, 75th percentile of 0.28, and interquartile range (IQR) of 0.07, indicating that many compounds in this external library were structurally distant from the training compounds. The empirical applicability-domain threshold, defined as the 5th percentile of the within-training nearest-neighbor Tanimoto similarity distribution, was 0.204. Among the 53 external compounds, 9 (17.0%) fell below this threshold, while 42 (79.2%) had nearest-neighbor similarities below 0.3; only 4 (7.5%) had similarities of 0.5 or higher, and none had similarities of 0.7 or higher.

**Table 4:** Performance metrics for external validation using the 53-compound library.

Performance metrics	Value
Sensitivity	0.0
Specificity	1.0
PPV	NA
NPV	0.62
Balanced accuracy	0.5
MCC	0
Accuracy	0.62
ROC-AUC	0.37
AP	0.32

**Fig 3:**
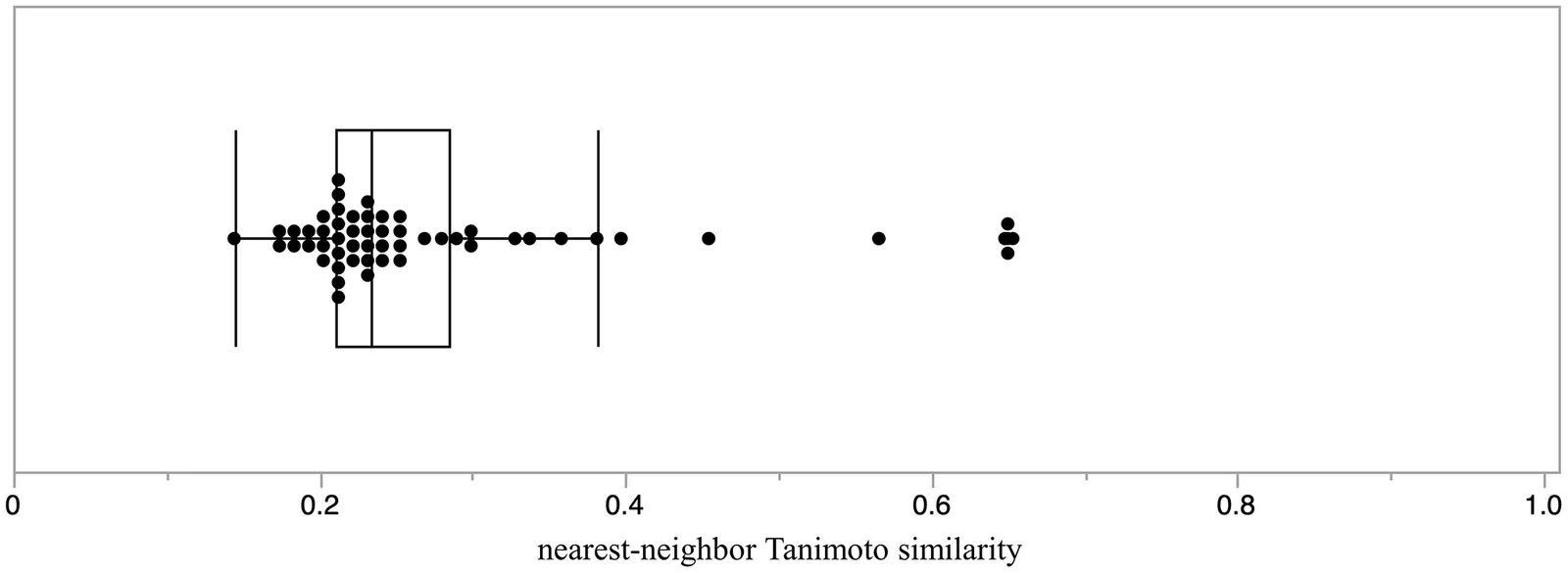
Distribution of nearest-neighbor (NN) Tanimoto similarity between the external validation compounds and the training set. NN Tanimoto similarity was calculated for each compound in the 53-compound external validation library using Morgan fingerprints (radius = 2, 1024 bits). For each external compound, the NN Tanimoto similarity was defined as the maximum Tanimoto similarity to any compound in the training set. The box plot summarizes the distribution of external compounds by NN Tanimoto similarity. Lower values indicate lower structural similarity to the training compounds, whereas higher values indicate greater structural proximity to the training chemical space. (Median of 0.23, 25th percentile of 0.21, 75th percentile of 0.28, and interquartile range (IQR) of 0.07).

We next tested whether prediction correctness in the external set could be explained by structural proximity to the training compounds (Table 5). Logistic regression analysis found no statistically significant association between nearest-neighbor Tanimoto similarity and prediction correctness (β = −4.545, *p* = 0.0865, pseudo-R² = 0.048). Among the 20 experimentally active compounds in the external set, none were predicted as active, including compounds with relatively high nearest-neighbor similarities to the training set. Therefore, a logistic regression model for active recovery as a function of nearest-neighbor similarity could not be fitted because all active compounds had the same recovery status. Prediction scores were also not significantly correlated with measured cell viability (Spearman’s ρ = 0.069, *p* = 0.6254) or experimental activity labels (Spearman’s ρ = −0.216, *p* = 0.1198).

**Table 5:** Association between structural proximity and external prediction performance.

Analysis	Outcome variable	Predictor/ variable compared	n	Estimate	*p*-value
Logistic regression	Prediction correctness	NN-Tanimoto similarity	53	β = −4.545; pseudo-R² = 0.048	0.0865
Spearman correlation	Measured cell viability	Prediction score	53	ρ = 0.069	0.6254
Spearman correlation	Experimental activity label	Prediction score	53	ρ = −0.216	0.1198

To examine whether the observed internal discrimination could be explained by random label assignment, we performed Y-randomization (Table 6). Using the original activity labels, the fixed cross-validation workflow yielded a ROC-AUC of 0.756. In contrast, 1000 random permutations of the labels produced a null ROC-AUC distribution centered near 0.5 (0.497 ± 0.062), with a maximum value of 0.679. The empirical *p*-value was 0.0010.

**Table 6:** Y-randomization analysis of internal model discrimination.

Analysis	Label condition	Number of permutations	ROC-AUC	Null AUC (±SD)	Max Null AUC	Empirical *p*-value
Fixed CV workflow	Original	NA	0.756	NA	NA	NA
Y-randomization	Randomly permuted	1000	NA	0.497 ± 0.062	0.679	0.0010

As an additional sensitivity analysis, we compared a random 5-folds cross-validation with a leave-scaffold-out grouped cross-validation based on Bemis–Murcko scaffolds. The training set contained 191 scaffold groups, and no scaffold group was shared between the training and test folds in the grouped cross-validation. The random 5-folds CV yielded a ROC-AUC of 0.758 (95% CI, 0.671–0.839), AP of 0.573, MCC of 0.423, and balanced accuracy of 0.712. The leave-scaffold-out CV yielded a similar ROC-AUC of 0.769 (95% CI, 0.696–0.841) and balanced accuracy of 0.721, although AP and MCC decreased to 0.434 and 0.354, respectively (Table 7).

**Table 7:** Comparison of random and scaffold-grouped cross-validation. The calculation was performed on all 236 compounds.

Analysis	Scaffold grouping	ROC-AUC	AP	MCC	BA	Sensitivity	Specificity
Random 5 folds-CV	No	0.758	0.573	0.423	0.712	0.533	0.890
Leave-scaffold-out CV	Yes	0.769	0.434	0.354	0.721	0.756	0.686

We next inspected the locked prediction scores on the external set to determine why no active compounds were recovered. The Brier score was 0.281, indicating substantial probabilistic prediction error on the external compounds. The highest predicted score in the external set was 0.662, which was below the predefined decision threshold of 0.708; therefore, no compound exceeded the threshold and all 53 compounds were classified as inactive.

## 4. Discussion

In this study, we used the experimental results for cell viability against U-251 cells accumulated in our laboratory to construct an activity classification model using machine learning.

Of the 236 compounds, 45 met the predefined activity criterion of <50% U-251 cell viability at the screening concentration of 10 µM or at a lower tested concentration. In contrast, 191 compounds did not meet the predefined activity criterion (cell viability ≥50%, Fig 1, S1 Table), resulting in a dataset containing a higher proportion of inactive compounds. The 236 compounds used in this study were primarily selected from an FDA-approved drug library, a collection well-suited for drug repositioning research, and covers a wide range of therapeutic areas, including oncology, immunology, and cardiovascular and neurological disorders. In addition, the set included several compounds, such as individually purchased anticancer and central nervous system drugs as well as compounds synthesized in our laboratory. In compound evaluation, the concentration at which 50% cell viability is observed, such as the IC_50_, is generally regarded as an important threshold. However, because the present study used single-point screening data, IC_50_ values could not be directly estimated for all compounds. Therefore, we defined active compounds operationally as those reducing U-251 cell viability to below 50% at or below the upper screening concentration of 10 µM, corresponding to more than 50% inhibition of cell viability relative to the vehicle control. This threshold provides an experimentally interpretable criterion for distinguishing compounds with marked in vitro activity under the screening condition and has also been used in previous machine-learning studies based on cell viability data [17,18]. Therefore, we adopted 50% cell viability as the threshold for classifying compounds.

To minimize optimistic bias and provide a reliable estimate of generalization performance for a small and imbalanced dataset, we adopted a repeated nested cross-validation framework in which hyperparameter tuning and feature selection were conducted strictly within the training folds. This leakage-controlled design allowed us to evaluate model performance across multiple independent outer test splits and to report metrics with uncertainty (mean ± 95% CI), rather than relying on a single “lucky” train/test split. In this context, the observed performance should be interpreted as a realistic estimate of what can be expected when applying the model to new compounds that are structurally similar to the training chemical space.

The terms “active” and “inactive” used in this study should be interpreted strictly as operational labels under the predefined screening framework using 10 µM as the upper screening concentration and a 48 h exposure period, rather than as measures of pharmacological potency or therapeutic suitability. A 10 µM cutoff has been widely used as a pragmatic threshold to define “active” versus “inactive” in large-scale bioactivity analyses and screening datasets, but such cutoffs are inherently heuristic and can depend on assay conditions, biological endpoints, and project-specific goals [19]. We therefore adopted 10 µM as a practical operational benchmark for an initial cell-based prioritization model rather than as a definitive pharmacological potency criterion. Because systematic concentration–response data were not available for all compounds, dose–response relationships could not be incorporated into the classification framework. In addition, matched cytotoxicity data in non-neoplastic cells were not available for the compound set; therefore, selectivity indices could not be calculated. Consequently, a compound classified as inactive in the present assay may still have pharmacological interest if it exhibits favorable potency or selectivity under more detailed testing, whereas a compound classified as active may represent nonspecific cytotoxicity rather than tumor-selective activity. At the same time, we acknowledge that, in the context of central nervous system (CNS) drug discovery, BBB-limited brain exposure can make 10 µM a relatively high screening concentration from a translational perspective. Importantly, an in vitro viability assay does not capture pharmacokinetic constraints such as BBB penetration or free drug concentrations in the brain. Accordingly, our “active” label should be interpreted as indicating cellular activity under the specified in vitro screening conditions, not guaranteed efficacy at clinically achievable CNS exposures. Future work will address these limitations by expanding dose–response measurements, evaluating lower concentrations and/or multiple concentration points, assessing cytotoxicity in non-neoplastic cells, and integrating BBB-related considerations (e.g., permeability/efflux or unbound brain exposure) into the prioritization framework.

In data-driven QSAR and machine-learning models, prediction reliability is generally constrained by the chemical and response space represented in the training data. The structural diversity alone does not guarantee reliable prediction for all compounds, but adequate structural diversity in the training set is an important foundation for constructing QSAR models with broader applicability. In addition, it is important to consider whether external validation compounds fall within the applicability domain of the model [20,21]. To assess the diversity of our dataset, we calculated Tanimoto similarity and Bemis–Murcko scaffolds based on ECFP (Fig 2 and Table 3). The structural diversity of the training set was supported by complementary similarity and scaffold-based analyses. Using ECFP (Morgan fingerprints; radius = 2, 1024 bits), the within-training NN Tanimoto similarity was generally low (median = 0.33; 25th–75th percentile = 0.26–0.54 (IQR = 0.28)), indicating that most compounds did not have very close analogs within the dataset. In parallel, Bemis–Murcko scaffold analysis showed a high scaffold-level diversity (189 unique scaffolds among 234 compounds; scaffold ratio = 0.81), with limited dominance of any single chemical series (Top-1 share = 0.06; Top-5 share = 0.15) and a highly even scaffold distribution (normalized Shannon entropy = 0.96). Taken together, these results indicate scaffold-level diversity and limited dominance of repeated scaffold series within the training set, which is desirable for developing and evaluating machine-learning models. At the same time, these measures reflect structural diversity and do not necessarily imply biological diversity; therefore, conclusions regarding generalization should be interpreted together with applicability-domain analyses and external validation.

In this study, binary predictions were obtained by applying a decision threshold to the model scores. As an operational choice, we used the Youden index–based threshold, which selects the cutoff that maximizes the sum of sensitivity and specificity (i.e., maximizes Youden’s J). This approach provides a commonly used, objective threshold when a single cutoff is required and when sensitivity and specificity are considered equally important. However, threshold-dependent metrics such as sensitivity, specificity, PPV/NPV, F1, balanced accuracy, and MCC can change substantially with the chosen cutoff. Therefore, the Youden threshold should be interpreted as one practical reference point rather than a universally optimal operating point.

The present dataset is moderately imbalanced, with substantially fewer active than inactive compounds, which can bias model training toward the majority class and reduce sensitivity for detecting actives. To mitigate this issue, we incorporated class weighting into the modeling workflow for algorithms that support it (e.g., logistic regression, SVM, random forest, and ExtraTrees). Specifically, the class_weight parameter was included as a hyperparameter and optimized within the inner loop of the nested cross-validation. This approach increases the penalty for misclassifying the minority (active) class during training and can improve minority-class recognition without altering the original data distribution. Despite this adjustment, performance gains may remain limited in small and noisy datasets or when external compounds are structurally distant from the training chemical space. Therefore, class weighting should be viewed as a practical mitigation strategy rather than a complete solution.

The poor external performance was consistent with a general shift away from the training chemical space, but the AD analysis did not support a simple nearest-neighbor Tanimoto explanation. Prediction correctness was not significantly improved by higher nearest-neighbor similarity, and all experimentally active external compounds were missed regardless of similarity bin. Therefore, a simple similarity-based applicability domain was insufficient to identify a subset of external compounds for which predictions were reliable.

The Y-randomization analysis provides evidence that the internal performance was not merely a consequence of random label assignment. After permutation of the activity labels, the cross-validated ROC-AUC values collapsed to a null distribution centered near 0.5, whereas the model trained with the original labels retained a higher ROC-AUC. This supports the presence of a non-random structure–activity signal within the in-house dataset. However, this result should not be interpreted as evidence of external generalizability. Indeed, the model failed to recover active compounds in the independent external set, indicating that the internal signal captured by the model did not transfer robustly to chemically distinct external compounds.

The leave-scaffold-out analysis provides additional context for interpreting the internal model performance. The ROC-AUC was not reduced relative to random cross-validation when Bemis–Murcko scaffolds were used as grouping labels, suggesting that the internal ranking performance was not solely driven by trivial scaffold leakage. However, AP and MCC were lower under scaffold-grouped cross-validation, and the dataset contained a large number of scaffold groups, many of which were represented by only one or a few compounds. Therefore, this analysis does not rule out scaffold- or chemotype-specific effects. Importantly, the preservation of internal ROC-AUC under scaffold-grouped cross-validation did not translate into successful external generalization, as the final model failed to recover active compounds in the independent external set. This discrepancy further emphasizes that internal validation, even with scaffold-aware splitting, is insufficient to establish external transferability in small chemical datasets.

The discrepancy between internal and external performance should be interpreted as evidence of limited transferability rather than as a failure of the validation workflow itself. The Y-randomization and scaffold-grouped cross-validation analyses suggest that the in-house dataset contained a non-random internal signal. However, the external validation results indicate that this signal was not sufficiently general to support reliable prediction for independent compounds. This finding underscores the need for larger and more structurally diverse active compound sets, predefined external validation designs, and careful applicability-domain assessment before such models are used for prospective compound prioritization.

Although the fixed decision threshold contributed directly to the absence of positive predictions in the external set, the probabilistic prediction and ranking analyses indicate a broader limitation. All external compounds received scores below the final threshold, and the external Brier score indicated substantial probabilistic prediction error. Moreover, the external ROC-AUC was below random expectation, indicating that lowering the threshold alone would not necessarily yield reliable prioritization. Thus, the external failure reflects both threshold-dependent classification collapse and insufficient transferability of the learned score ranking.

The external set showed evidence of covariate shift relative to the training set, as reflected by lower nearest-neighbor Tanimoto similarities and the scarcity of close analogs. Nevertheless, a simple nearest-neighbor similarity threshold was insufficient to account for the external failure. Most external compounds were not classified as outside the empirical applicability-domain threshold, and prediction correctness was not significantly associated with nearest-neighbor similarity. These findings indicate that structural distance from the training set was an important contextual factor, but not a sufficient explanation for the loss of external performance.

In conclusion, using a corrected, single-center dataset of 236 compounds generated under a standardized experimental protocol (45 active and 191 inactive compounds), we developed a binary classifier for U-251 cell-viability activity using a leakage-controlled repeated nested cross-validation framework. In this workflow, preprocessing, feature selection, and hyperparameter optimization were performed strictly within the training folds, and ExtraTrees was selected based on Matthews correlation coefficient. The model showed moderate internal discrimination, with an internal ROC-AUC of approximately 0.76, BA of 0.66 and MCC of 0.36. However, when the locked final model was applied to an independent external set of 53 structurally distinct compounds, it classified all compounds as inactive, resulting in zero sensitivity, balanced accuracy of 0.5, and MCC of 0. The external ROC-AUC based on continuous prediction scores was 0.37, indicating that the external failure was not explained solely by the fixed classification threshold. Moreover, the hypothesis that prediction errors were determined by structural distance from the training set was not supported by nearest-neighbor Tanimoto similarity analysis. Thus, the main contribution of this study is not the development of a ready-to-use screening tool, but rather the provision of a reusable corrected dataset and reproducible modeling pipeline, together with a cautionary generalization-gap benchmark showing that favorable internal cross-validation performance may not transfer beyond the local chemical space represented in a small, imbalanced training dataset. Future work will focus on expanding the dataset, particularly by increasing the number and structural diversity of active compounds, to reduce class imbalance and support the development of more practically useful models for identifying active compounds.

## Supporting information

S1 File Compound preparation, synthesis, and characterization of the synthesized compounds. (DOCX)

S2 File ^1^H NMR, ^13^C NMR, and MS spectra of the synthesized compounds. (DOCX)

S1 Table U-251 cell viability data for the 236 compounds used for model development. (XLSX)

S2 Table Aggregated confusion matrices across all outer test folds. (DOCX)

S3 Table Data for the 53 compounds used for independent external evaluation. (XLSX)

## References

[1] Ostrom QT, Price M, Neff C, Cioffi G, Waite KA, Kruchko C, et al. CBTRUS statistical report: Primary brain and other central nervous system tumors diagnosed in the United States in 2015-2019. Neuro Oncol. 2022;24(Suppl 5):v1–95. doi: 10.1093/neuonc/noac202 36196752 PMC9533228

[2] Pandit R, Chen L, Götz J. The blood-brain barrier: Physiology and strategies for drug delivery. Adv Drug Deliv Rev. 2020;165–166:1–14. doi: 10.1016/j.addr.2019.11.009 31790711

[3] Zhang J, Stevens MFG, Bradshaw TD. Temozolomide: Mechanisms of action, repair and resistance. Curr Mol Pharmacol. 2012;5(1):102–14. doi: 10.2174/1874467211205010102 22122467

[4] Lee SY. Temozolomide resistance in glioblastoma multiforme. Genes Dis. 2016;3(3):198–210. doi: 10.1016/j.gendis.2016.04.007 30258889 PMC6150109

[5] Pushpakom S, Iorio F, Eyers PA, Escott KJ, Hopper S, Wells A, et al. Drug repurposing: progress, challenges and recommendations. Nat Rev Drug Discov. 2019;18(1):41–58. doi: 10.1038/nrd.2018.168 30310233

[6] Siegelin MD, Schneider E, Westhoff M-A, Wirtz CR, Karpel-Massler G. Current state and future perspective of drug repurposing in malignant glioma. Semin Cancer Biol. 2021;68:92–104. doi: 10.1016/j.semcancer.2019.10.018 31734137

[7] Tsuchiya N, Okamoto K, Nakao S, Ohmori S, Shimizu T. Effect of Blonanserin on the Proliferation and Migration of Glioblastoma Cells. Pharmazie. 2023;78(5):37–41. doi: 10.1691/ph.2023.1821 37189270

[8] Uemichi Y, Mabuchi M, Tsuchiya N, Yasuda S, Nakao S, Shimizu T. Effects of Lomerizine and Its Metabolite on Glioblastoma Cells. Anticancer Res. 2024;44(7):2943–52. doi: 10.21873/anticanres.17106 38925834

[9] Abbas MKG, Rassam A, Karamshahi F, Abunora R, Abouseada M. The Role of AI in drug discovery. Chembiochem. 2024;25(14):e202300816. doi: 10.1002/cbic.202300816 38735845

[10] Chan HCS, Shan H, Dahoun T, Vogel H, Yuan S. Advancing Drug Discovery via Artificial Intelligence. Trends Pharmacol Sci. 2019;40(8):592–604. doi: 10.1016/j.tips.2019.06.004 31320117

[11] Miao Y, Ma H, Huang J. Recent advances in toxicity prediction: Applications of deep graph learning. Chem Res Toxicol. 2023;36(8):1206–26. doi: 10.1021/acs.chemrestox.2c00384 37562046

[12] Sinha K, Ghosh N, Sil PC. A Review on the Recent Applications of Deep Learning in Predictive Drug Toxicological Studies. Chem Res Toxicol. 2023;36(8):1174–205. doi: 10.1021/acs.chemrestox.2c00375 37561655

[13] Kurosaki K, Wu R, Uesawa Y. A Toxicity Prediction Tool for Potential Agonist/Antagonist Activities in Molecular Initiating Events Based on Chemical Structures. Int J Mol Sci. 2020;21(21):7853. doi: 10.3390/ijms21217853 33113912 PMC7660166

[14] Mendez D, Gaulton A, Bento AP, Chambers J, De Veij M, Félix E, et al. ChEMBL: towards direct deposition of bioassay data. Nucleic Acids Res. 2019;47(D1):D930–40. doi: 10.1093/nar/gky1075 30398643 PMC6323927

[15] Wang Y, Bryant SH, Cheng T, Wang J, Gindulyte A, Shoemaker BA, et al. PubChem bioassay: 2017 update. Nucleic Acids Res. 2017;45(D1):D955–63. doi: 10.1093/nar/gkw1118 27899599 PMC5210581

[16] Neves BJ, Agnes JP, Gomes M do N, Henriques Donza MR, Gonçalves RM, Delgobo M, et al. Efficient identification of novel anti-glioma lead compounds by machine learning models. Eur J Med Chem. 2020;189:111981. doi: 10.1016/j.ejmech.2019.111981 31978780

[17] Ariey-Bonnet J, Berges R, Montero M-P, Mouysset B, Piris P, Muller K, et al. Combination drug screen targeting glioblastoma core vulnerabilities reveals pharmacological synergisms. EBioMedicine. 2023;95:104752. doi: 10.1016/j.ebiom.2023.104752 37572644 PMC10433015

[18] Wu Y, Tang K, Wang C, Song H, Zhou F, Guo Y. Establishment of interpretable cytotoxicity prediction models using machine learning analysis of transcriptome features. Acta Pharm Sin B. 2025;15(3):1344–58. doi: 10.1016/j.apsb.2025.02.009 40370539 PMC12069252

[19] López-López E, Fernández-de Gortari E, Medina-Franco JL. Yes SIR! On the structure-inactivity relationships in drug discovery. Drug Discov Today. 2022;27(8):2353–62. doi: 10.1016/j.drudis.2022.05.005 35561964

[20] Golbraikh A, Muratov E, Fourches D, Tropsha A. Data set modelability by QSAR. J Chem Inf Model. 2014;54(1):1–4. doi: 10.1021/ci400572x 24251851 PMC3984298

[21] Sahigara F, Mansouri K, Ballabio D, Mauri A, Consonni V, Todeschini R. Comparison of Different Approaches to Define the Applicability Domain of QSAR Models. Molecules. 2012;17(5):4791–810. doi: 10.3390/molecules17054791 22534664 PMC6268288

