# Supplementary material for: Rigorous nested cross-validation and external generalization-gap analysis of a machine-learning classifier for U-251 glioblastoma cytotoxicity
Source: PLoS One. 2026 Nov 3;21(11):e0361912. doi: 10.1371/journal.pone.0361912 (PMC13730374; doi:10.1371/journal.pone.0361912)
Supplement: Supplementary file 1 [file pone.0361912.s001.docx]

**Supporting Information**

**Rigorous nested cross-validation and external generalization-gap analysis of a machine-learning classifier for U-251 glioblastoma cytotoxicity**

Yuki Uemichi^a^, Shuhei Nakao^a*^, Shuji Nagano^a^, Miyuki Mabuchi^a^, Yuya Kawabata^a^, Yoshihiro Uesawa^b^, Tadashi Shimizu^a^

^a^ School of Pharmacy, Hyogo Medical University, Kobe, Hyogo, Japan

^b^ Department of Medical Molecular Informatics, Meiji Pharmaceutical University, Kiyose, Tokyo, Japan

**Chemistry**

**Compound preparation**

The following compounds were purchased commercially: Tocriscreen FDA-Approved Drug Library (Fujifilm Wako, 7200, Osaka-shi, Osaka, Japan), Paclitaxel (Tokyo Chemical Industry (TCI), P1632, Chuo-ku, Tokyo, Japan), Ledipasvir (Merck, AMBH2D6FA54E, Darmstadt, Germany), Darunavir (Merck, AMBH47A8ED73, Darmstadt, Germany), Bezafibrate (TCI, B3346, Chuo-ku, Tokyo, Japan), Podophyllotoxin (TCI, P1771, Chuo-ku, Tokyo, Japan), Daunorubicin (Fujifilm Wako, 049-31241, Osaka-shi, Osaka, Japan), Nortriptyline (TCI, N0957, Chuo-ku, Tokyo, Japan), Vinblastine (Fujifilm Wako, 224-02321, Osaka-shi, Osaka, Japan), Vinorelbine (Fujifilm Wako, 222-01641, Osaka-shi, Osaka, Japan), Vincristine (TCI, V0129, Chuo-ku, Tokyo, Japan), Colchicine (Fujifilm Wako, 039-03851, Osaka-shi, Osaka, Japan), Vindesine (MedChemExpress, HY-129071, New Jersey, USA), Topotecan (TOCRIS, 4562, Abingdon, UK), Mometasone (TCI, M2354, Chuo-ku, Tokyo, Japan), Hemin (TCI, H0008, Chuo-ku, Tokyo, Japan), Azelastine (TCI, A2340, Chuo-ku, Tokyo, Japan), Lomerizine (TCI, L0245, Chuo-ku, Tokyo, Japan), Blonanserin (TCI, B4565, Chuo-ku, Tokyo, Japan), Cinnarizine (TCI, C3459, Chuo-ku, Tokyo, Japan), Flunarizine (Merck, F8257, Darmstadt, Germany), Verapamil hydrochloride (TCI, V0118, Chuo-ku, Tokyo, Japan), 1-(bis(4-fluorophenyl)methyl)piperazine (TCI, B2764, Chuo-ku, Tokyo, Japan).

The 53 compounds used in the external evaluation were purchased commercially (Selleck Biotechnology, custom compound library, Kanagawa, Japan). Details are provided in Table S3.

The following compounds were synthesized according to a previously reported procedure, and their structure were confirmed by comparison with the reported spectral data: FK614,^1)^ OPC-14857,^2)^ 2,3,4,5-tetrahydro-1H-pyrido[4,3-b]indole-6-carboxylic acid,^3)^ 4-(4-fluorophenyl)-2-(piperazin-1-yl)-5,6,7,8,9,10-hexahydrocycloocta[b]pyridine,^4)^ 1-phenyl-3-(5-(trifluoromethyl)-1H-benzo[d]imidazol-2-yl) imidazolidin-2-one.^5)^

**Synthesis procedure**

**(4-(bis(4-fluorophenyl)methyl)piperazin-1-yl)(phenyl)methanone (YK-001)**

To a solution of 1-[bis(4-fluorophenyl)methyl]piperazine (200 mg, 0.69 mmol) in pyridine (2.0 mL) was added benzoyl chloride (117 mg, 0.83 mmol). The mixture was stirred for 1.5 h at room temperature, and then water was added. The aqueous layer was extracted with ethyl acetate. The combined organic layers were washed with water and brine, and the organic layer was dried over MgSO_4_. After filtration to remove MgSO₄, the organic layer was concentrated using a rotary evaporator. The residue was purified by silica gel column chromatography (hexane/ethyl acetate = 4:1) to give the desired compound (161 mg, 59%) as a white solid. ^1^H-NMR (400 MHz, CDCl_3_) δ 2.30 (s, 2H), 2.45 (s, 2H), 3.42 (s, 2H), 3.78 (s, 2H), 4.25 (s, 1H), 6.94-7.01 (m, 4H), 7.31-7.40 (m, 9H); ^13^C-NMR (100 MHz, CDCl_3_) δ 42.3, 51.7, 52.2, 74.4, 115.2-116.4 (m), 126.7-127.5 (m), 128.1-130.1 (m), 135.8, 137.7, 160.8, 163.2, 170.3; HRMS (ESI^+^) calcd. for C_24_H_23_F_2_N_2_O ([M+H]^+^) m/z 393.1778; found 393.1781.

**1-(4-(2,3-dichlorophenyl)piperazin-1-yl)butan-1-one (YK-002)**

To a solution of 1-(2,3-dichlorophenyl)piperazine (200 mg, 0.87 mmol) in pyridine (2.0 mL) was added butanoyl chloride (91.7 mg, 0.87 mmol). The mixture was stirred for 1.5 h at room temperature, and then water was added. The aqueous layer was extracted with ethyl acetate. The combined organic layers were washed with water and brine, and the organic layer was dried over MgSO_4_. After filtration to remove MgSO₄, the organic layer was concentrated using a rotary evaporator. The residue was purified by silica gel column chromatography (hexane/ethyl acetate = 1:1) to give the desired compound (142 mg, 47%) as a colorless oil. ^1^H-NMR (400 MHz, CDCl_3_) δ 0.99 (t, *J* = 7.4 Hz, 3H), 1.58-1.74 (sext, *J* = 7.6 Hz, 2H), 2.36 (t, *J* = 7.6 Hz, 2H), 2.99-3.04 (m, 4H), 3.65 (dd, J = 5.0 Hz, 2H), 3.80 (dd, *J* = 4.4 Hz, 2H), 6.92 (dd, *J* = 8.0 and 1.6 Hz, 1H), 7.14-7.22 (m, 2H); ^13^C-NMR (100 MHz, CDCl_3_) δ 14.1, 18.9, 35.4, 41.7, 45.9, 51.3, 51.8, 118.6-119.1 (m), 124.8-125.7 (m), 127.4-128.1 (m), 134.2, 150.7, 171.8; HRMS (ESI^+^) calcd. for C_14_H_19_Cl_2_N_2_O ([M+H]^+^) m/z 301.0874; found 301.0857.

**1-(bis(4-fluorophenyl)methyl)-4-(3,4-dichlorobenzyl)piperazine (YK-003)**

To a solution of 1-[bis(4-fluorophenyl)methyl]piperazine (640 mg, 2.2 mmol) in DMF (7.0 mL) was added 3,4-dichlorobenzyl bromide (906 mg, 3.8 mmol) and K_2_CO_3_ (614 mg, 4.4 mmol). The mixture was stirred for 66 h at 50 °C, and then water was added. The aqueous layer was extracted with ethyl acetate. The combined organic layers were washed with water and brine, and the organic layer was dried over MgSO_4_. After filtration to remove MgSO₄, the organic layer was concentrated using a rotary evaporator. The residue was purified by silica gel column chromatography (hexane/ethyl acetate = 9:1) to give the desired compound (675 mg, 68%) as a yellow oil. ^1^H-NMR (400 MHz, CDCl_3_) δ 2.45 (br, 8H), 3.45 (s, 2H), 4.22 (s, 1H), 6.92-6.99 (m, 4H), 7.13 (dd, *J* = 2.2 and 8.4 Hz, 1H), 7.31-7.37 (m, 5H), 7.40 (d, *J* = 2.0 Hz, 1H); ^13^C-NMR (100 MHz, CDCl_3_) δ 51.7, 53.3, 61.7, 74.5, 115.2-115.8 (m), 128.3-130.9 (m), 131.0, 132.4, 138.2, 138.6, 160.7, 163.1; HRMS (ESI^+^) calcd. for C_24_H_23_Cl_2_F_2_N_2_ ([M+H]^+^) m/z 447.1206; found 447.1202.

**1-benzhydryl-4-(2,3,4-trimethoxybenzyl)piperazine (YK-005)**

To a solution of diphenylmethyl mesylate (98 mg, 0.37 mmol) in DMF (1.0 mL) was added 1-(2,3,4-trimethoxybenzyl)piperazine (71 mg, 0.27 mmol) and K_2_CO_3_ (86 mg, 0.62 mmol). The mixture was stirred for 18 h at room temperature, and then KI (10 mg, 0.062 mmol) was added. The mixture was stirred for 2.5 h at 50 °C, and then water was added. The aqueous layer was extracted with ethyl acetate. The combined organic layers were washed with water and brine, and the organic layer was dried over MgSO_4_. After filtration to remove MgSO₄, the organic layer was concentrated using a rotary evaporator. The residue was purified by silica gel column chromatography (hexane/ethyl acetate = 3:1) to give the desired compound (11 mg, 9.6%) as a yellow oil. ^1^H-NMR (400 MHz, CDCl_3_); δ 2.50 (br, 8H), 3.49 (s, 2H), 3.84 (s, 3H), 3.86 (s, 3H), 3.87 (s, 3H), 4.2 (s, 1H), 6.61 (d, *J* = 8.4 Hz, 1H), 6.97 (d, *J* = 8.8 Hz, 1H), 7.14-7.18 (m, 2H), 7.24 (d, *J* = 8.0 Hz, 2H), 7.26 (d, *J* = 6.8 Hz, 2H), 7.38-7.42 (m, 4H); ^13^C-NMR (100 MHz, CDCl_3_) δ 52.1, 53.2, 56.0, 56.4, 60.9, 61.3, 107.0, 125.4, 126.9, 128.0, 128.5, 142.3, 143.0, 152.7, 152.9; HRMS (ESI^+^) calcd. for C_27_H_33_N_2_O_3_ ([M+H]^+^) m/z 433.2491; found 433.2493.

**1-(bis(4-fluorophenyl)methyl)-4-(methylsulfonyl)piperazine (YK-006)**

To a solution of 1-[bis(4-fluorophenyl)methyl]piperazine (200 mg, 0.69 mmol) in CHCl_3_ (2.0 mL) was added methanesulfonyl chloride (119 mg, 1.0 mmol) and triethylamine (140 mg, 1.4 mmol) in an ice bath. The mixture was stirred for 8 h at 50 °C, and then water was added. The aqueous layer was extracted with ethyl acetate. The combined organic layers were washed with water and brine, and the organic layer was dried over MgSO_4_. After filtration to remove MgSO₄, the organic layer was concentrated using a rotary evaporator. The residue was purified by silica gel column chromatography (hexane/ethyl acetate = 3:2) to give the desired compound (152 mg, 60%) as a white solid. ^1^H-NMR (400 MHz, CDCl_3_); δ 2.48 (dd, *J* = 4.8 Hz, 4H), 2.79 (s, 3H), 3.24 (dd, *J* = 4.8 Hz, 4H), 4.28 (s, 1H), 6.96-7.03 (m, 4H), 7.31-7.37 (m, 4H); ^13^C-NMR (100 MHz, CDCl_3_) δ 33.9-34.3 (m), 46.2, 51.0 (t, *J* = 8.4 Hz), 74.0 (dd, *J* = 2.4 Hz), 115.5-116.1 (m), 129.3-129.5 (m), 137.57, 137.60, 160.8, 163.2, HRMS (ESI^+^) calcd. for C_18_H_20_F_2_N_2_NaO_2_S ([M+Na]^+^) m/z 389.1111; found 389.1100.

**1-(4-fluorobenzyl)-4-(2,3,4-trimethoxybenzyl)piperazine (YK-007)**

To a solution of 1-(2,3,4-trimethoxybenzyl)piperazine (100 mg, 0.38 mmol) in CHCl_3_ (1.0 mL) was added 4-fluorobenzyl bromide (125 mg, 0.66 mmol) and triethylamine (89 mg, 0.88 mmol) in an ice bath. The mixture was stirred for 3.5 h at room temperature, and then water was added. The aqueous layer was extracted with ethyl acetate. The combined organic layers were washed with water and brine, and the organic layer was dried over MgSO_4_. After filtration to remove MgSO₄, the organic layer was concentrated using a rotary evaporator. Hexane was added to the residue, followed by filtration. The filtrate was concentrated to afford the desired compound (100 mg, 71%) as a yellow oil. ^1^H-NMR (400 MHz, CDCl_3_); δ 2.47 (br, 8H), 3.45 (s, 2H), 3.48 (s, 2H), 3.84 (s, 3H), 3.86 (s, 3H), 3.87 (s, 3H), 6.62 (d, *J* = 8.8 Hz, 1H), 6.95-7.01 (m, 3H), 7.23-7.28 (m, 2H); ^13^C-NMR (100 MHz, CDCl_3_) δ 52.9, 53.2, 55.9-56.1 (m), 56.5, 60.9, 61.3, 62.3, 106.8-107.0 (m), 114.4-115.6 (m), 124.0, 124.9-125.0 (m), 125.4-125.8 (m), 130.1-131.2 (m), 133.9-134.0 (m), 142.3, 152.7 (d, *J* = 2.9 Hz), 152.9 (d, *J* = 2.9 Hz), 160.8, 163.2; HRMS (ESI^+^) calcd. for C_21_H_28_FN_2_O_3_ ([M+H]^+^) m/z 375.2084; found 375.2069.

**1-(2,3,4-trifluorobenzyl)-4-(2,3,4-trimethoxybenzyl)piperazine (YK-008)**

To a solution of 1-(2,3,4-trimethoxybenzyl)piperazine (100 mg, 0.38 mmol) in CHCl_3_ (1.0 mL) was added 1-(bromomethyl)-2,3,4-trifluorobenzene (199 mg, 0.88 mmol) and triethylamine (112 mg, 1.1 mmol) in an ice bath. The mixture was stirred for 72 h at room temperature, and then water was added. The aqueous layer was extracted with ethyl acetate. The combined organic layers were washed with water and brine, and the organic layer was dried over MgSO_4_. After filtration to remove MgSO₄, the organic layer was concentrated using a rotary evaporator. The residue was purified by silica gel column chromatography (CHCl_3_/MeOH = 19:1 to 9:1) to give the desired compound (27 mg, 18%) as a colorless oil. ^1^H-NMR (400 MHz, CDCl_3_); δ 2.49 (br, 8H), 3.48 (s, 2H), 3.54 (s, 2H), 3.84 (s, 3H), 3.86 (s, 3H), 3.87 (s, 3H), 6.62 (d, *J* = 8.4 Hz, 1H), 6.87-6.94 (m, 1H), 6.96 (d, *J* = 8.4 Hz, 1H), 7.04-7.11 (m, 1H); ^13^C-NMR (100 MHz, CDCl_3_) δ 52.8, 52.9, 54.8, 56.0-56.1 (m), 56.5, 60.9, 61.3, 106.9-107.0 (m), 122.4, 122.5, 123.9, 124.9-125.1 (m), 125.5-125.6 (m), 142.3, 152.7 (d, *J* = 2.9 Hz), 152.9 (d, *J* = 2.9 Hz); HRMS (ESI^+^) calcd. for C_21_H_26_F_3_N_2_O_3_ ([M+H]^+^) m/z 411.1896; found 411.1901.

**phenyl(4-(2,3,4-trimethoxybenzyl)piperazin-1-yl)methanone (YK-009)**

To a solution of 1-(2,3,4-trimethoxybenzyl)piperazine (100 mg, 0.38 mmol) in CHCl_3_ (1.0 mL) was added benzoyl chloride (75 mg, 0.53 mmol) and triethylamine (67 mg, 0.66 mmol) in an ice bath. The mixture was stirred for 7 h at room temperature, and then water was added. The aqueous layer was extracted with CHCl_3_. The combined organic layers were washed with water and brine, and the organic layer was dried over MgSO_4_. After filtration to remove MgSO₄, the organic layer was concentrated using a rotary evaporator. The residue was purified by silica gel column chromatography (CHCl_3_) to give the desired compound (143 mg, quant.) as a colorless oil. ^1^H-NMR (400 MHz, CDCl_3_); δ 2.41 (br, 2H), 2.56 (br, 2H), 3.41 (br, 2H), 3.51 (s, 2H), 3.78 (br, 2H), 3.85 (s, 3H), 3.87 (s, 3H), 3.88 (s, 3H), 6.64 (d, *J* = 8.4 Hz, 1H), 6.97 (d, *J* = 8.8 Hz, 1H), 7.38-7.41 (m, 5H); ^13^C-NMR (100 MHz, CDCl_3_) δ 42.3, 47.9, 52.3-53.4 (m), 55.9-56.2 (m), 56.5, 60.8-61.0 (m), 61.2-61.4 (m), 107.1, 123.3, 125.0-126.0 (m), 127.0-127.7 (m), 128.0-129.0 (m), 129.5-130.0 (m), 135.9, 142.3, 152.7, 153.2, 170.4; HRMS (ESI^+^) calcd. for C_21_H_27_N_2_O_4_ ([M+H]^+^) m/z 371.1971; found 371.1975.

**(2-chlorophenyl)(4-(2,3,4-trimethoxybenzyl)piperazin-1-yl)methanone (YK-010)**

To a solution of 1-(2,3,4-trimethoxybenzyl)piperazine (100 mg, 0.38 mmol) in CHCl_3_ (1.0 mL) was added 2-chlorobenzoyl chloride (92 mg, 0.53 mmol) and triethylamine (67 mg, 0.66 mmol) in an ice bath. The mixture was stirred for 23 h at room temperature, and then water was added. The aqueous layer was extracted with CHCl_3_. The combined organic layers were washed with water and brine, and the organic layer was dried over MgSO_4_. After filtration to remove MgSO₄, the organic layer was concentrated using a rotary evaporator. The residue was purified by silica gel column chromatography (ethyl acetate/hexane = 4:1) to give the desired compound (99 mg, 65%) as a colorless oil. ^1^H-NMR (400 MHz, CDCl_3_); δ 2.30-2.37 (m, 1H), 2.44-2.63 (m, 3H), 3.14-3.31 (m, 2H), 3.49 (s, 2H), 3.78-3.83 (m, 2H), 3.84 (s, 3H), 3.86 (s, 3H), 3.88 (s, 3H), 6.63 (d, *J* = 8.8 Hz, 1H), 6.96 (d, *J* = 8.4 Hz, 1H), 7.25-7.34 (m, 3H), 7.37-7.40 (m, 1H); ^13^C-NMR (100 MHz, CDCl_3_) δ 41.8, 46.9, 52.1-53.4 (m), 55.9-56.2 (m), 56.4-56.6 (m), 60.8-61.0 (m), 61.2-61.4 (m), 107.0, 123.4, 125.0-125.5 (m), 126.9-128.1 (m), 129.5-130.3 (m), 130.4, 135.9, 142.3, 152.7, 153.2, 166.9; HRMS (ESI^+^) calcd. for C_21_H_26_ClN_2_O_4_ ([M+H]^+^) m/z 405.1581; found 405.1588.

**(4-chlorophenyl)(4-(2,3,4-trimethoxybenzyl)piperazin-1-yl)methanone (YK-011)**

To a solution of 1-(2,3,4-trimethoxybenzyl)piperazine (100 mg, 0.38 mmol) in CHCl_3_ (1.0 mL) was added 4-chlorobenzoyl chloride (92 mg, 0.53 mmol) and triethylamine (67 mg, 0.66 mmol) in an ice bath. The mixture was stirred for 23 h at room temperature, and then water was added. The aqueous layer was extracted with CHCl_3_. The combined organic layers were washed with water and brine, and the organic layer was dried over MgSO_4_. After filtration to remove MgSO₄, the organic layer was concentrated using a rotary evaporator. The residue was purified by silica gel column chromatography (ethyl acetate/hexane = 4:1) to give the desired compound (63 mg, 41%) as a colorless oil. ^1^H-NMR (400 MHz, CDCl_3_); δ 2.41 (br, 2H), 2.55 (br, 2H), 3.40 (br, 2H), 3.50 (s, 2H), 3.76 (br, 2H), 3.85 (s, 3H), 3.87 (s, 3H), 3.88 (s, 3H), 6.64 (d, *J* = 8.4 Hz, 1H), 6.97 (d, *J* = 8.4 Hz, 1H), 7.32-7.39 (m, 4H); ^13^C-NMR (100 MHz, CDCl_3_) δ 42.3, 47.9, 52.0-53.5 (m), 55.9-56.2 (m), 56.3-56.6 (m), 60.8-61.0 (m), 61.2-61.4 (m), 107.1, 123.2, 125.1-125.5 (m), 128.5-129.0 (m), 134.2, 135.8, 142.3, 152.7, 153.2, 169.3; HRMS (ESI^+^) calcd. for C_21_H_26_ClN_2_O_4_ ([M+H]^+^) m/z 405.1581; found 405.1589.

**1-(2-fluorobenzyl)-4-(2,3,4-trimethoxybenzyl)piperazine (YK-012)**

To a solution of 1-(2,3,4-trimethoxybenzyl)piperazine (50 mg, 0.19 mmol) in THF (1.0 mL) was added 2-fluorobenzyl bromide (46 mg, 0.24 mmol) and triethylamine (34 mg, 0.33 mmol) in an ice bath. The mixture was stirred for 4.5 h at room temperature, and then water was added. The aqueous layer was extracted with ethyl acetate. The combined organic layers were washed with water and brine, and the organic layer was dried over MgSO_4_. After filtration to remove MgSO₄, the organic layer was concentrated using a rotary evaporator. The residue was purified by silica gel column chromatography (ethyl acetate/hexane = 4:1) to give the desired compound (30 mg, 41%) as a yellow oil. ^1^H-NMR (400 MHz, CDCl_3_); δ 2.51 (br, 8H), 3.48 (s, 2H), 3.59 (s, 2H), 3.84 (s, 3H), 3.86 (s, 3H), 3.87 (s, 3H), 6.62 (d, *J* = 8.4 Hz, 1H), 6.97 (d, *J* = 8.4 Hz, 1H), 6.98-7.03 (m, 1H), 7.08 (td, *J* = 7.4 and 1.2 Hz, 1H), 7.19-7.25 (m, 1H), 7.35 (td, *J* = 7.1 and 1.6 Hz, 1H); ^13^C-NMR (100 MHz, CDCl_3_) δ 52.9, 55.2-55.5 (m), 55.9-56.2 (m), 56.5, 60.8-61.0 (m), 61.1-61.5 (m), 107.0, 124.0, 124.6, 124.8, 125.2, 125.3, 125.4, 142.3, 152.7, 152.9, 160.3, 162.7; HRMS (ESI^+^) calcd. for C_21_H_27_FN_2_O_3_Na ([M+Na]^+^) m/z 397.1903; found 397.1895.

**(E)-3-(benzo[d][1,3]dioxol-5-yl)-1-(4-(2,3,4-trimethoxybenzyl)piperazin-1-yl)prop-2-en-1-one (YK-013)**

To a solution of 1-(2,3,4-trimethoxybenzyl)piperazine (200 mg, 0.75 mmol) in CHCl_3_ (6.0 mL) was added (2E)-3-(1,3-benzodioxol-5-yl)-2-propenoic acid (221 mg, 1.1 mmol), 1-hydroxybenzotriazole (155 mg, 1.1 mmol), 1-(3-dimethylaminopropyl)-3-ethylcarbodiimide hydrochloride (220 mg, 1.1 mmol) in an ice bath. The mixture was stirred for 69 h at room temperature, and then water was added. The aqueous layer was extracted with ethyl acetate. The combined organic layers were washed with sat. NaHCO_3_aq, 1 M HCl, water and brine, and the organic layer was dried over MgSO_4_. After filtration to remove MgSO₄, the organic layer was concentrated using a rotary evaporator. The residue was purified by silica gel column chromatography (CHCl_3_) to give the desired compound (187 mg, 57%) as a colorless oil. ^1^H-NMR (400 MHz, CDCl_3_); δ 2.49 (dd, *J* = 4.8 Hz, 4H), 3.50 (s, 2H), 3.62 (br, 2H), 3.72 (br, 2H), 3.86 (s, 3H), 3.87 (s, 3H), 3.89 (s, 3H), 5.99 (s, 2H), 6.65 (d, *J* = 8.8 Hz, 1H), 6.69 (d, *J* = 15.6 Hz, 1H), 6.80 (s, *J* = 8.0 Hz, 1H), 6.98-7.03 (m, 3H), 7.57 (d, *J* = 15.2 Hz, 1H); ^13^C-NMR (100 MHz, CDCl_3_) δ 42.3, 46.0, 52.4-53.5 (m), 55.9-56.2 (m), 56.5, 60.8-61.0 (m), 61.2-61.4 (m), 101.5, 106.2, 106.6, 107.0, 115.2, 123.4, 129.8, 142.4, 148.3, 149.0, 152.7, 153.2, 165.5; HRMS (ESI^+^) calcd. for C_24_H_29_N_2_O_6_ ([M+H]^+^) m/z 441.2026; found 441.2002.

**2-(5-methoxy-1H-indol-3-yl)-1-(4-(2,3,4-trimethoxybenzyl)piperazin-1-yl)ethan-1-one (YK-014)**

To a solution of 1-(2,3,4-trimethoxybenzyl)piperazine (100 mg, 0.38 mmol) in DMF (3.0 mL) was added 5-methoxyindoleacetic acid (118 mg, 0.58 mmol), 1-hydroxybenzotriazole (78 mg, 0.58 mmol), 1-(3-dimethylaminopropyl)-3-ethylcarbodiimide hydrochloride (110 mg, 0.58 mmol) in an ice bath. The mixture was stirred for 26 h at room temperature, and then water was added. The aqueous layer was extracted with ethyl acetate. The combined organic layers were washed with water and brine, and the organic layer was dried over MgSO_4_. After filtration to remove MgSO₄, the organic layer was concentrated using a rotary evaporator. The residue was purified by silica gel column chromatography (CHCl_3_) to give the desired compound (84 mg, 49%) as an orange solid. ^1^H-NMR (400 MHz, CDCl_3_); δ 2.28 (dd, *J* = 4.6 Hz, 2H), 2.43 (dd, *J* = 4.8 Hz, 2H), 3.42 (s, 2H), 3.45 (dd, *J* = 5.0 Hz, 2H), 3.65 (dd, *J* = 4.6 Hz, 2H), 3.78 (s, 2H), 3.84 (s, 9H), 3.85 (s, 3H), 6.61 (d, *J* = 8.4 Hz, 1H), 6.83 (dd, *J* = 8.8 and 2.4 Hz, 1H), 6.92 (d, *J* = 8.4 Hz, 1H), 6.98 (d, *J* = 2.4 Hz, 1H), 7.03 (d, *J* = 2.8 Hz, 1H), 7.21 (d, *J* = 8.8 Hz, 1H), 8.37 (br, 1H); ^13^C-NMR (100 MHz, CDCl_3_) δ 31.5, 41.9, 46.3, 52.0-53.1 (m), 55.9-56.2 (m), 56.4, 60.8-61.0 (m), 61.2-61.4 (m), 100.3, 100.7, 109.0, 111.9-112.4 (m), 123.4, 127.5, 131.4, 142.3, 152.7, 154.1, 170.2; HRMS (ESI^+^) calcd. for C_25_H_32_N_3_O_5_ ([M+H]^+^) m/z 454.2342; found 454.2314.

**2-((4-(2,3,4-trimethoxybenzyl)piperazin-1-yl)methyl)quinoline (YK-015)**

To a solution of 1-(2,3,4-trimethoxybenzyl)piperazine (58 mg, 0.22 mmol) in CH_3_CN (0.70 mL) was added 2-(chloromethyl)quinoline (50 mg, 0.23 mmol) and Na_2_CO_3_ (74 mg, 0.70 mmol) in an ice bath. The mixture was stirred at 70 °C for 3.5 h, and then water was added. The aqueous layer was extracted with ethyl acetate. The combined organic layers were washed with water and brine, and the organic layer was dried over MgSO_4_. After filtration to remove MgSO₄, the organic layer was concentrated using a rotary evaporator. The residue was purified by silica gel column chromatography (CHCl_3_/MeOH = 19:1) to give the desired compound (47 mg, 52%) as a brown oil. ^1^H-NMR (400 MHz, CDCl_3_); δ 2.58 (br, 8H), 3.50 (s, 2H), 3.83 (s, 2H), 3.84 (s, 3H), 3.86 (s, 3H), 3.87 (s, 3H), 6.62 (d, *J* = 8.4 Hz, 1H), 6.98 (d, *J* = 8.4 Hz, 1H), 7.47-7.53 (m, 1H), 7.62 (d, *J* = 8.8 Hz, 1H), 7.65-7.71 (m, 1H), 7.79 (d, *J* = 8.0 Hz, 1H), 8.06 (d, *J* = 8.8 Hz, 1H), 8.11 (d, *J* = 8.4 Hz, 1H); ^13^C-NMR (100 MHz, CDCl_3_) δ 52.8-54.0 (m), 56.0-56.1 (m), 56.4, 60.8-61.0 (m), 61.3, 65.2, 106.9-107.1 (m), 121.3, 123.9, 126.2, 127.5, 127.6, 129.1, 129.5, 142.3, 147.7, 152.69, 152.73, 152.92, 152.95, 159.7; HRMS (ESI^+^) calcd. for C_24_H_30_N_3_O_3_ ([M+H]^+^) m/z 408.2287; found 408.2261.

**1-(4-chlorobenzyl)-4-(2,3,4-trimethoxybenzyl)piperazine (YK-016)**

To a solution of 1-(2,3,4-trimethoxybenzyl)piperazine (50 mg, 0.19 mmol) in THF (0.5 mL) was added 4-chlorobenzyl bromide (50 mg, 0.24 mmol) and triethylamine (45 mg, 0.44 mmol) in an ice bath. The mixture was stirred for 24 h at room temperature, and then water was added. The aqueous layer was extracted with ethyl acetate. The combined organic layers were washed with water and brine, and the organic layer was dried over MgSO_4_. After filtration to remove MgSO₄, the organic layer was concentrated using a rotary evaporator. The residue was purified by silica gel column chromatography (CHCl_3_/MeOH = 19:1) to give the desired compound (50 mg, 67%) as a yellow oil. ^1^H-NMR (400 MHz, CDCl_3_); δ 2.46 (br, 8H), 3.45 (s, 2H), 3.48 (s, 2H), 3.84 (s, 3H), 3.86 (s, 3H), 3.87 (s, 3H), 6.62 (d, *J* = 8.4 Hz, 1H), 6.97 (d, *J* = 8.4 Hz, 1H), 7.22-7.28 (m, 4H); ^13^C-NMR (100 MHz, CDCl_3_) δ 52.9, 53.2, 56.0, 56.5, 60.9, 61.3, 62.4, 106.8-107.1 (m), 124.0, 124.9-125.6 (m), 127.8-129.0 (m), 130.0-131.1 (m), 132.7, 136.9, 142.3, 152.69, 152.73, 152.90, 152.94; HRMS (ESI^+^) calcd. for C_21_H_28_ClN_2_O_3_ ([M+H]^+^) m/z 391.1788; found 391.1802.

**(1H-imidazol-5-yl)(4-(2,3,4-trimethoxybenzyl)piperazin-1-yl)methanone (YK-017)**

To a solution of 1-(2,3,4-trimethoxybenzyl)piperazine (100 mg, 0.38 mmol) in DMF (1.3 mL) was added 1H-imidazole-5-carboxylic acid (64 mg, 0.58 mmol), 1-hydroxybenzotriazole (84 mg, 0.62 mmol), 1-(3-dimethylaminopropyl)-3-ethylcarbodiimide hydrochloride (110 mg, 0.58 mmol) and triethylamine (157 mg, 1.5 mmol) in an ice bath. The mixture was stirred for 99 h at room temperature, and then water was added. The aqueous layer was extracted with ethyl acetate. The combined organic layers were washed with water and brine, and the organic layer was dried over MgSO_4_. After filtration to remove MgSO₄, the organic layer was concentrated using a rotary evaporator. The residue was purified by silica gel column chromatography (CHCl_3_/MeOH = 9:1) to give the desired compound (55 mg, 40%) as a colorless oil. ^1^H-NMR (400 MHz, CDCl_3_); δ 2.54 (dd, *J* = 4.8 Hz, 2H), 3.51 (s, 2H), 3.61-3.64 (m, 1H), 3.73-3.80 (m, 2H), 3.85 (s, 3H), 3.87 (s, 3H), 3.88 (s, 3H), 4.00-4.18 (m, 1H), 6.64 (d, *J* = 8.8 Hz, 1H), 6.98 (d, *J* = 8.4 Hz, 1H), 7.43 (br, 1H), 7.61 (br, 1H); ^13^C-NMR (100 MHz, CDCl_3_) δ 42.9, 47.0, 52.0-54.0 (m), 55.9-56.2 (m), 56.5, 60.8-61.0 (m), 61.2-61.5 (m), 72.4, 107.1, 123.3, 125.0-125.7 (m), 142.3, 152.69, 152.72, 153.15, 153.17; HRMS (ESI^+^) calcd. for C_18_H_25_N_4_O_4_ ([M+H]^+^) m/z 361.1876; found 361.1878.

**1-(2,4-dichlorobenzyl)-4-(2,3,4-trimethoxybenzyl)piperazine (YK-018)**

To a solution of 1-(2,3,4-trimethoxybenzyl)piperazine (50 mg, 0.19 mmol) in THF (0.75 mL) was added 2,4-dichlorobenzyl chloride (48 mg, 0.25 mmol) and diisopropylethylamine (57 mg, 0.44 mmol) in an ice bath. The mixture was stirred for 48 h at room temperature, and then water was added. The aqueous layer was extracted with ethyl acetate. The combined organic layers were washed with water and brine, and the organic layer was dried over MgSO_4_. After filtration to remove MgSO₄, the organic layer was concentrated using a rotary evaporator. The residue was purified by silica gel column chromatography (CHCl_3_/MeOH = 19:1) to give the desired compound (29 mg, 36%) as a colorless oil. ^1^H-NMR (400 MHz, CDCl_3_); δ 2.51 (br, 8H), 3.48 (s, 2H), 3.56 (s, 2H), 3.85 (s, 3H), 3.87 (s, 3H), 3.88 (s, 3H), 6.63 (d, *J* = 8.4 Hz, 1H), 6.98 (d, *J* = 8.4 Hz, 1H), 7.20 (dd, *J* = 8.2, 2.4 Hz, 1H), 7.35 (d, *J* = 2.0 Hz, 1H), 7.41 (d, *J* = 8.4 Hz, 1H); ^13^C-NMR (100 MHz, CDCl_3_) δ 53.0, 53.3, 55.9-56.2 (m), 56.5, 58.7, 60.8-61.0 (m), 61.3, 107.0, 124.0, 125.0-125.5 (m), 126.5-127.2 (m), 128.9-129.9 (m), 131.2-132.0 (m), 133.0, 134.8, 134.9, 142.3, 152.70, 152.73, 152.92, 152.94; HRMS (ESI^+^) calcd. for C_21_H_27_Cl_2_N_2_O_3_ ([M+H]^+^) m/z 425.1399; found 425.1410.

**1-(4-methylbenzyl)-4-(2,3,4-trimethoxybenzyl)piperazine (YK-019)**

To a solution of 1-(2,3,4-trimethoxybenzyl)piperazine (50 mg, 0.19 mmol) in THF (0.75 mL) was added 4-methylbenzyl chloride (34 mg, 0.24 mmol) and diisopropylethylamine (57 mg, 0.44 mmol) in an ice bath. The mixture was stirred for 53 h at 50 °C, and then water was added. The aqueous layer was extracted with ethyl acetate. The combined organic layers were washed with water and brine, and the organic layer was dried over MgSO_4_. After filtration to remove MgSO₄, the organic layer was concentrated using a rotary evaporator. The residue was purified by silica gel column chromatography (ethyl acetate) to give the desired compound (33 mg, 47%) as a colorless oil. ^1^H-NMR (400 MHz, CDCl_3_); δ 2.32 (s, 3H), 2.48 (br, 8H), 3.45 (s, 2H), 3.48 (s, 2H), 3.84 (s, 3H), 3.86 (s, 3H), 3.87 (s, 3H), 6.62 (d, *J* = 8.4 Hz, 1H), 6.97 (d, *J* = 8.4 Hz, 1H), 7.11 (d, *J* = 7.6 Hz, 2H), 7.18 (d, *J* = 8.0 Hz, 2H); ^13^C-NMR (100 MHz, CDCl_3_) δ 21.2, 52.9, 53.2, 56.0, 56.5, 60.9, 61.3, 62.9, 106.8-107.0 (m), 124.1, 124.8-125.6 (m), 128.4-130.0 (m), 135.1, 136.7, 142.3, 152.70, 152.73, 152.88, 152.91; HRMS (ESI^+^) calcd. for C_22_H_31_N_2_O_3_ ([M+H]^+^) m/z 371.2355; found 371.2344.

**thiophen-2-yl(4-(2,3,4-trimethoxybenzyl)piperazin-1-yl)methanone (YK-020)**

To a solution of 1-(2,3,4-trimethoxybenzyl)piperazine (50 mg, 0.19 mmol) in CHCl_3_ (0.50 mL) was added 2-thiophenecarbonyl chloride (36 mg, 0.24 mmol) and triethylamine (34 mg, 0.33 mmol) in an ice bath. The mixture was stirred for 3 h at room temperature, and then water was added. The aqueous layer was extracted with ethyl acetate. The combined organic layers were washed with water and brine, and the organic layer was dried over MgSO_4_. After filtration to remove MgSO₄, the organic layer was concentrated using a rotary evaporator. The residue was purified by silica gel column chromatography (ethyl acetate/hexane = 4:1) to give the desired compound (19 mg, 27%) as a colorless oil. ^1^H-NMR (400 MHz, CDCl_3_); δ 2.53 (br, 4H), 3.52 (s, 2H), 3.75 (dd, *J* = 4.8 Hz, 4H), 3.86 (s, 3H), 3.87 (s, 3H), 3.89 (s, 3H), 6.64 (d, *J* = 8.4 Hz, 1H), 6.98 (d, *J* = 8.4 Hz, 1H), 7.03 (dd, *J* = 5.2 and 3.6 Hz, 1H), 7.27 (d, *J* = 0.8 Hz, 1H), 7.43 (dd, *J* = 5.2 and 1.2 Hz, 1H); ^13^C-NMR (100 MHz, CDCl_3_) δ 52.9, 55.9-56.2 (m), 56.4, 60.8-61.0 (m), 61.2-61.4 (m), 107.1, 123.1, 125.0-125.6 (m), 126.2-127.0 (m), 128.1-129.1 (m), 137.2, 142.4, 152.7, 153.2, 163.6; HRMS (ESI^+^) calcd. for C_19_H_25_N_2_O_4_S ([M+H]^+^) m/z 377.1535; found 377.1543.

**1-(2-chlorobenzyl)-4-(2,3,4-trimethoxybenzyl)piperazine (YK-021)**

To a solution of 1-(2,3,4-trimethoxybenzyl)piperazine (100 mg, 0.38 mmol) in THF (1.5 mL) was added 2-chlorobenzyl bromide (100 mg, 0.49 mmol) and triethylamine (112 mg, 1.1 mmol) in an ice bath. The mixture was stirred for 23 h at room temperature, and then water was added. The aqueous layer was extracted with ethyl acetate. The combined organic layers were washed with water and brine, and the organic layer was dried over MgSO_4_. After filtration to remove MgSO₄, the organic layer was concentrated using a rotary evaporator. The residue was purified by silica gel column chromatography (CHCl_3_/MeOH = 49:1) to give the desired compound (68 mg, 46%) as a yellow oil. ^1^H-NMR (400 MHz, CDCl_3_); δ 2.53 (s, 8H), 3.49 (s, 2H), 3.61 (s, 2H), 3.85 (s, 3H), 3.87 (s, 3H), 3.88 (s, 3H), 6.63 (d, *J* = 8.8 Hz, 1H), 6.89 (d, *J* = 8.8 Hz, 1H), 7.14-7.25 (m, 2H), 7.33 (dd, *J* = 7.8 and 1.2 Hz, 1H), 7.46 (dd, *J* = 7.6 and 1.6 Hz, 1H); ^13^C-NMR (100 MHz, CDCl_3_) δ 53.0, 53.3, 55.9-56.1 (m), 56.5, 59.3, 60.9, 61.3, 106.8-107.1 (m), 124.1, 124.4-130.0 (m), 130.8, 134.4, 136.0, 142.3, 152.70, 152.73, 152.90, 152.93; HRMS (ESI^+^) calcd. for C_21_H_28_ClN_2_O_3_ ([M+H]^+^) m/z 391.1788; found 391.1770.

**1-(3-chlorobenzyl)-4-(2,3,4-trimethoxybenzyl)piperazine (YK-022)**

To a solution of 1-(2,3,4-trimethoxybenzyl)piperazine (100 mg, 0.38 mmol) in CHCl_3_ (1.0 mL) was added 3-chlorobenzyl bromide (100 mg, 0.49 mmol) and triethylamine (112 mg, 1.1 mmol) in an ice bath. The mixture was stirred for 17.5 h at room temperature, and then water was added. The aqueous layer was extracted with ethyl acetate. The combined organic layers were washed with water and brine, and the organic layer was dried over MgSO_4_. After filtration to remove MgSO₄, the organic layer was concentrated using a rotary evaporator. The residue was purified by silica gel column chromatography (ethyl acetate) to give the desired compound (77 mg, 52%) as a colorless oil. ^1^H-NMR (400 MHz, CDCl_3_); δ 2.47 (br, 8H), 3.46 (s, 2H), 3.48 (s, 2H), 3.84 (s, 3H), 3.86 (s, 3H), 3.87 (s, 3H), 6.62 (d, *J* = 8.8 Hz, 1H), 6.97 (d, *J* = 8.8 Hz, 1H), 7.16-7.23 (m, 3H), 7.32 (s, 1H); ^13^C-NMR (100 MHz, CDCl_3_) δ 52.9, 53.2, 55.9-56.2 (m), 56.5, 60.8-61.0 (m), 61.2-61.4 (m), 62.5, 107.0, 123.9, 125.3-125.4 (m), 127.3, 129.1, 129.2, 129.5, 134.2, 140.5, 142.3, 152.7, 152.9; HRMS (ESI^+^) calcd. for C_21_H_28_ClN_2_O_3_ ([M+H]^+^) m/z 391.1788; found 391.1759.

**1-(4-(trifluoromethyl)benzyl)-4-(2,3,4-trimethoxybenzyl)piperazine (YK-023)**

To a solution of 1-(2,3,4-trimethoxybenzyl)piperazine (100 mg, 0.38 mmol) in THF (1.5 mL) was added 4-(trifluoromethyl)benzyl bromide (116 mg, 0.49 mmol) and triethylamine (112 mg, 1.1 mmol) in an ice bath. The mixture was stirred for 21 h at room temperature, and then water was added. The aqueous layer was extracted with ethyl acetate. The combined organic layers were washed with water and brine, and the organic layer was dried over MgSO_4_. After filtration to remove MgSO₄, the organic layer was concentrated using a rotary evaporator. The residue was purified by silica gel column chromatography (ethyl acetate) to give the desired compound (107 mg, 66%) as a colorless oil. ^1^H-NMR (400 MHz, CDCl_3_); δ 2.48 (br, 8H), 3.49 (s, 2H), 3.54 (s, 2H), 3.84 (s, 3H), 3.86 (s, 3H), 3.87 (s, 3H), 6.62 (d, *J* = 8.8 Hz, 1H), 6.97 (d, *J* = 8.4 Hz, 1H), 7.43 (d, *J* = 8.0 Hz, 2H), 7.55 (d, *J* = 8.0 Hz, 2H); ^13^C-NMR (100 MHz, CDCl_3_) δ 52.9, 53.3, 55.9-56.2 (m), 56.5, 60.8-61.0 (m), 61.2-61.4 (m), 62.5, 107.0, 123.0, 123.8, 124.6-126.0 (m), 128.6-130.0 (m), 142.3, 142.7, 152.7, 153.0; HRMS (ESI^+^) calcd. for C_22_H_27_F_3_N_2_NaO_3_ ([M+Na]^+^) m/z 447.1871; found 447.1853.

**1-(4-nitrobenzyl)-4-(2,3,4-trimethoxybenzyl)piperazine (YK-024)**

To a solution of 1-(2,3,4-trimethoxybenzyl)piperazine (100 mg, 0.38 mmol) in THF (1.5 mL) was added 4-nitrobenzyl bromide (105 mg, 0.49 mmol) and triethylamine (112 mg, 1.1 mmol) in an ice bath. The mixture was stirred for 23 h at room temperature, and then water was added. The aqueous layer was extracted with ethyl acetate. The combined organic layers were washed with water and brine, and the organic layer was dried over MgSO_4_. After filtration to remove MgSO₄, the organic layer was concentrated using a rotary evaporator. The residue was purified by silica gel column chromatography (ethyl acetate) to give the desired compound (99 mg, 65%) as a yellow oil. ^1^H-NMR (400 MHz, CDCl_3_); δ 2.48 (br, 8H), 3.50 (s, 2H), 3.57 (s, 2H), 3.84 (s, 3H), 3.86 (s, 3H), 3.87 (s, 3H), 6.62 (d, *J* = 8.4 Hz, 1H), 6.97 (d, *J* = 8.8 Hz, 1H), 7.49 (d, *J* = 8.8 Hz, 2H), 8.14-8.18 (m, 2H); ^13^C-NMR (100 MHz, CDCl_3_) δ 52.8, 53.3, 55.9-56.2 (m), 56.4, 60.8-61.0 (m), 61.2-61.4 (m), 62.2, 107.0, 123.2-124.0 (m), 125.0-125.8 (m), 129.2-130.0 (m), 142.3, 146.5, 147.2, 152.7, 153.0; HRMS (ESI^+^) calcd. for C_21_H_28_N_3_O_5_ ([M+H]^+^) m/z 402.2029; found 402.2007.

**1-(4-methoxybenzyl)-4-(2,3,4-trimethoxybenzyl)piperazine (YK-025)**

To a solution of 1-(2,3,4-trimethoxybenzyl)piperazine (100 mg, 0.38 mmol) in THF (1.0 mL) was added 4-methoxybenzyl chloride (76 mg, 0.49 mmol) and triethylamine (112 mg, 1.1 mmol) in an ice bath. The mixture was stirred for 25 h at room temperature, and then water was added. The aqueous layer was extracted with ethyl acetate. The combined organic layers were washed with water and brine, and the organic layer was dried over MgSO_4_. After filtration to remove MgSO₄, the organic layer was concentrated using a rotary evaporator. The residue was purified by silica gel column chromatography (ethyl acetate) to give the desired compound (65 mg, 44%) as a colorless oil. ^1^H-NMR (400 MHz, CDCl_3_); δ 2.48 (br, 8H), 3.43 (s, 2H), 3.48 (s, 2H), 3.79 (s, 3H), 3.84 (s, 3H), 3.86 (s, 3H), 3.87 (s, 3H), 6.61 (d, *J* = 8.8 Hz, 1H), 6.82-6.86 (m, 2H), 6.97 (d, *J* = 8.4 Hz, 1H), 7.19-7.23 (m, 2H); ^13^C-NMR (100 MHz, CDCl_3_) δ 52.8, 53.1, 55.2-55.5 (m), 55.9-56.2 (m), 56.5, 60.7-61.0 (m), 61.2-61.3 (m), 62.5, 107.0, 112.0-115.0 (m), 123.9, 125.0-126.0 (m), 130.1, 130.4, 130.7, 142.3, 152.7, 152.9, 158.8; HRMS (ESI^+^) calcd. for C_22_H_31_N_2_O_4_ ([M+H]^+^) m/z 387.2284; found 387.2281.

**1-(bis(4-fluorophenyl)methyl)-4-tosylpiperazine (YK-029)**

To a solution of 1-[bis(4-fluorophenyl)methyl]piperazine (200 mg, 0.69 mmol) in CHCl_3_ (2.0 mL) was added tosyl chloride (159 mg, 0.83 mmol) and triethylamine (211 mg, 2.1 mmol). The mixture was stirred for 28 h at room temperature, and then water was added. The aqueous layer was extracted with ethyl acetate. The combined organic layers were washed with water and brine, and the organic layer was dried over MgSO_4_. After filtration to remove MgSO₄, the organic layer was concentrated using a rotary evaporator. The residue was purified by silica gel column chromatography (hexane/ethyl acetate = 4:1) to give the desired compound (280 mg, 91%) as a white solid. ^1^H-NMR (400 MHz, CDCl_3_); δ 2.43 (dd, *J* = 4.8 Hz, 2H), 2.47 (s, 3H), 3.00 (br, 4H), 4.20 (s, 1H), 6.89-6.96 (m, 4H), 7.22-7.28 (m, 4H), 7.35 (d, *J* = 8.0 Hz, 2H), 7.60-7.66 (m, 2H); ^13^C-NMR (100 MHz, CDCl_3_) δ 21.6-21.8 (m), 46.3, 50.9, 74.0, 114.7-116.4 (m), 127.7-128.5 (m), 128.7-130.5 (m), 132.4, 137.7, 143.8, 160.7, 163.2; HRMS (ESI^+^) calcd. for C_24_H_25_F_2_N_2_O_2_S ([M+H]^+^) m/z 443.1605, found 443.1599.

**References**

1. N. Yamasaki, T. Imoto, Y. Murai, *et al*., INDOLE DERIVATIVES, WO98/15530, April 16, 1998.

2. B. Satyanarayana, Y. Sumalatha, S. S. Kurmar, *et al.*, Synthesis and characterization of N-oxides and metabolites of anti-psychotic drug, aripiprazole. *Heterocyclic Communications,* 2005; 11 (6); 485-490. doi: 10.1515/HC.2005.11.6.485

3. A. J. Robichaud, J. M. Fevig, I. S. Mitchell, *et al.*, SUBSTITUTED PYRIDOINDOLES AS SEROTONIN AGONISTS AND ANTAGONISTS. US 2004/0186094 A1, September 23, 2004.

4. K. S. Rao, K.N. Rao, T. U. S. Sastry, *et al*., An investigation into formation of impurities during synthesis of blonanserin. *Asian Journal of Chemistry*, 2014; 26 (18); 5928-5930. doi: 10.14233/ajchem.2014.16331

5. C. Martin C., G. William J., C. Samuel, *et al.*, HETEROCYCLIC COMPOUNDS AS FACTOR IXA INHIBITORS. WO 2009/143039 A2, November 26, 2009.
