# Supplementary material for: Rigorous nested cross-validation and external generalization-gap analysis of a machine-learning classifier for U-251 glioblastoma cytotoxicity
Source: PLoS One. 2026 Nov 3;21(11):e0361912. doi: 10.1371/journal.pone.0361912 (PMC13730374; doi:10.1371/journal.pone.0361912)
Supplement: Supplementary file 2 [file pone.0361912.s002.docx]

^1^H, ^13^C NMR, and MS spectra

1) YK-001

2) YK-002

3) YK-003

4) YK-005

The integral appears larger due to overlap with the residual CH_3_Cl peak.

5) YK-006

6) YK-007

The integral appears larger due to overlap with the residual CH_3_Cl peak.

7) YK-008

8) YK-009

9) YK-010

The integral appears larger due to overlap with the residual CH_3_Cl peak.

10) YK-011

11) YK-012

12) YK-013

13) YK-014

14) YK-015

15) YK-016

The integral appears larger due to overlap with the residual CH_3_Cl peak.

16) YK-017

17) YK-018

18) YK-019

19) YK-020

The integral overlap with the residual CH_3_Cl peak.

20) YK-021

21) YK-022

22) YK-023

23) YK-024

24) YK-025

25) YK-029

The integral appears larger due to overlap with the residual CH_3_Cl peak.
