# Supplementary material for: Rigorous nested cross-validation and external generalization-gap analysis of a machine-learning classifier for U-251 glioblastoma cytotoxicity
Source: PLoS One. 2026 Nov 3;21(11):e0361912. doi: 10.1371/journal.pone.0361912 (PMC13730374; doi:10.1371/journal.pone.0361912)
Supplement: Supplementary file 3 [file pone.0361912.s004.docx]

S2 Table. The aggregated confusion matrices across all outer test folds (5 folds x 10 repeat)

|  | Negative  (Predicted label) | Positive  (Predicted label) |
| --- | --- | --- |
| Negative  (Experimental label) | 1725 | 185 |
| Positive  (Experimental label) | 264 | 186 |
